# Robust and Reprocessable Biorenewable Polyester Nanocomposites In Situ Catalyzed and Reinforced by Dendritic MXene@CNT Heterostructure

**DOI:** 10.1007/s40820-025-01682-8

**Published:** 2025-02-24

**Authors:** Hao Wang, Jiheng Ding, Hongran Zhao, Qinchao Chu, Jin Zhu, Jinggang Wang

**Affiliations:** 1 Key Laboratory of Bio-Based Polymeric Materials Technology and Application of Zhejiang Province, Ningbo Institute of Materials Technology and Engineering, Chinese Academy of Sciences, Ningbo, 315201 People’s Republic of China; 2 School of Materials Science and Chemical Engineering, Ningbo University, Ningbo, 315211 Zhejiang People’s Republic of China

**Keywords:** Bio-based polyesters nanocomposites, Dendritic hetero-structured MXene@CNT, Catalysis-interfacial strengthening integration, High strength and toughness, Reprocessability and multifunctionality

## Abstract

Renewable 2,5-furandicarboxylic acid-based polyesters are one of the most promising materials for achieving plastic replacement in the age of energy and environmental crisis. However, their properties still cannot compete with those of petrochemical-based plastics, owing to insufficient molecular and/or microstructure designs. Herein, we utilize the Ti_3_C_2_T_x_-based MXene nanosheets for decorating carbon nanotube (CNT) and obtaining the structurally stable and highly dispersed dendritic hetero-structured MXene@CNT, that can act as multi-roles, i.e., polycondensation catalyst, crystal nucleator, and interface enhancer of polyester. The bio-based MXene@CNT/polybutylene furandicarboxylate (PBF) (denoted as MCP) nanocomposites are synthesized by the strategy of “in situ catalytic polymerization and hot-pressing”. Benefiting from the multi-scale interactions (i.e., covalent bonds, hydrogen bonds, and physical interlocks) in hybrid structure, the MCP presents exceptional mechanical strength (≈101 MPa), stiffness (≈3.1 GPa), toughness (≈130 MJ m^−3^), and barrier properties (e.g., O_2_ 0.0187 barrer, CO_2_ 0.0264 barrer, and H_2_O 1.57 × 10^−14^ g cm cm^−2^ s Pa) that are higher than most reported bio-based materials and engineering plastics. Moreover, it also displays satisfactory multifunctionality with high reprocessability (90% strength retention after 5 recycling), UV resistance (blocking 85% UVA rays), and solvent-resistant properties. As a state-of-art high-performance and multifunctional material, the novel bio-based MCP nanocomposite offers a more sustainable alternative to petrochemical-based plastics in packaging and engineering material fields. More importantly, our catalysis-interfacial strengthening integration strategy opens a door for designing and constructing high-performance bio-based polyester materials in future.

**Supplementary Information:**

The online version contains supplementary material available at 10.1007/s40820-025-01682-8.

## Highlights

- Structurally stable and well-dispersed dendritic MXene@CNT heterostructures with multiple roles (i.e., catalyst, nucleator, and interface enhancer of polyesters) were constructed.
- Biorenewable MXene@CNT/PBF (MCP) polyester nanocomposites with ultrahigh mechanical strength (≈101 MPa), stiffness (≈3.1 GPa), and toughness (≈130 MJ m^−3^) were synthesized via MXene@CNT in situ catalytic polymerization.
- Exceptional reprocessability, gas barrier (e.g., O_2_ 0.0187 barrer), and UV resistance (e.g., resist 85% UVA rays) properties were achieved for the MCP, which can be employed as high-performance and multifunctional packaging materials for plastic replacement.

**Supplementary Information:**

The online version contains supplementary material available at 10.1007/s40820-025-01682-8.

## Graphical abstract

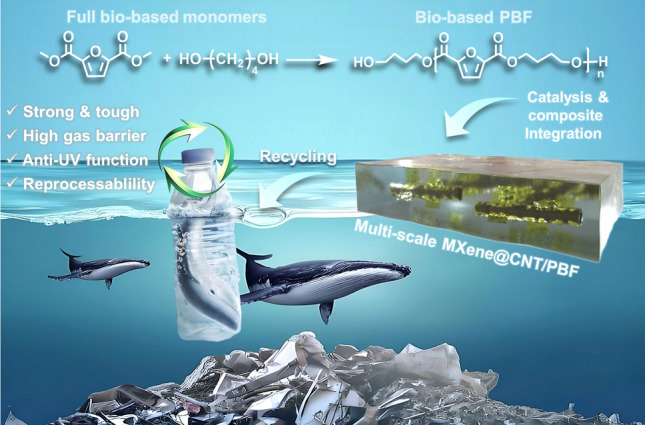

## Introduction

Plastics are inexpensive and serviceable materials. They are ubiquitously used in packaging, construction, electronics, and aviation [1]. Among plastics, polyethylene terephthalate (PET) is the most prolific petrochemical-based plastic, popularly utilized as a packaging material owing to its low density, water and gas barrier, and ease of manufacture [2]. However, most petrochemical-based plastics (e.g., PET, polyethylene [PE], polypropylene [PP], and polycarbonate [PC]) are nonbiodegradable because of their stable polymer backbones [3] and difficult to recycle and reuse [4, 5]. More than 100 million tons of plastic packaging wastes were generated every year around the world, one-third of which will end up in soils or oceans [6]. Plastic pollution will cause permanent damage to the environment and highly negative influence on human health. Therefore, it is vitally important to developing bio-based sustainable materials for achieving petrochemical-based plastic substitution.

According to the U.S. Department of Energy, 2,5-furandicarboxylic acid (FDCA) is one of the most promising building blocks for bio-based materials and the substantial performance components to compete with petrochemical-based plastics (e.g., aromatic PET and PC) [7]. FDCA is commonly applied to synthesize polyester packaging materials showing superior barrier properties owing to the domain-limited flipping and polar feature of furan rings as well as the ability to participate in hydrogen bonds [8]. Currently, FDCA-based polyesters are an emerging and promising packaging materials for achieving the sustainability and recyclability of plastics, of which the synthesis of FDCA-based homopolyesters (e.g., polyethylene furandicarboxylate [PEF], polypropylene furandicarboxylate [PPF], and polybutylene furandicarboxylate [PBF], etc.) [2, 9–11] and copolyesters (e.g., polybutylene carbonate-cofurandicarboxylate [PBCF] and polypropylene succinate-co-furandicarboxylate [PPSF]) [12, 13] have shown tremendous progress. However, the fabrication and application of FDCA-based polyester materials still face two key challenges: One is the lack of efficient polycondensation catalysts, and the polycondensation mechanism is still unclear; another is the lack of principles and methods to achieve performances upgrades, making it difficult to balance strength, toughness, and barrier properties.

Nanocomposite strategy is an advanced interface reinforcement approach to endow polymers with extraordinary joint increments in mechanical and functional properties [14, 15]. As typical 1D carbon nanofiber materials, carbon nanotube (CNT) is famous for its unparalleled mechanical prowess, such as ultrahigh fracture strength (≈100 GPa) and elastic modulus (≈950 GPa) [16]. Besides, CNT can act as a nano-nucleator to promote crystallization and enhance barrier properties of polymers, making it an ideal functional filler for constructing advanced FDCA-based polyester nanocomposites. However, CNT has not fulfilled this promise, mainly due to its poor dispersion and weak interface interactions with matrix [17, 18]. Several classical approaches (e.g., surfactants attachments, molecule and polymer grafting) have been explored to enhance CNT dispersion and mitigate the interfacial problems [19, 20]; nevertheless, to some extent, the trade-off was the decrease of its intrinsic characteristics and additional costs [21]. Ti_3_C_2_T_x_ (MXene), a burgeoning 2D nanomaterial, on account of its eminent physical and mechanical performance [22, 23], has attracted widespread scientific attentions. The easy exfoliation and dispersion characteristics make at-scale fabrication of MXene nanosheets at fast and low cost, boosting its actual applications [23–25]. The abundant surface functional groups (T_x_, i.e., –F, =O, and –OH) and high specific areas enable MXene to be the superior interface agent to improve the filler**-**matrix bridge through multiple interface interactions (e.g., covalent bonds [19, 20], electrostatic interaction [21], and hydrogen bonds [22]). Moreover, MXene also shows potential catalytic activity, owing to its distinctive surface natures and tunable Lewis/ Brönsted acidities [23, 24]. For example, a recent report on the catalysis of PET synthesis based on Ti_3_C_2_T_x_-based MXene revealed that the MXene could replace heavy metal catalysts to achieve the melt polycondensation reaction of PET [25]. Unfortunately, due to strong interlayer van der Waals forces, MXene nanosheets also face the problem of agglomeration [26], coupled with its high susceptibility to oxidation and deterioration [27, 28], all of which inevitably decreases MXene catalytic and mechanical enhancement efficiencies.

**Fig. 1:**
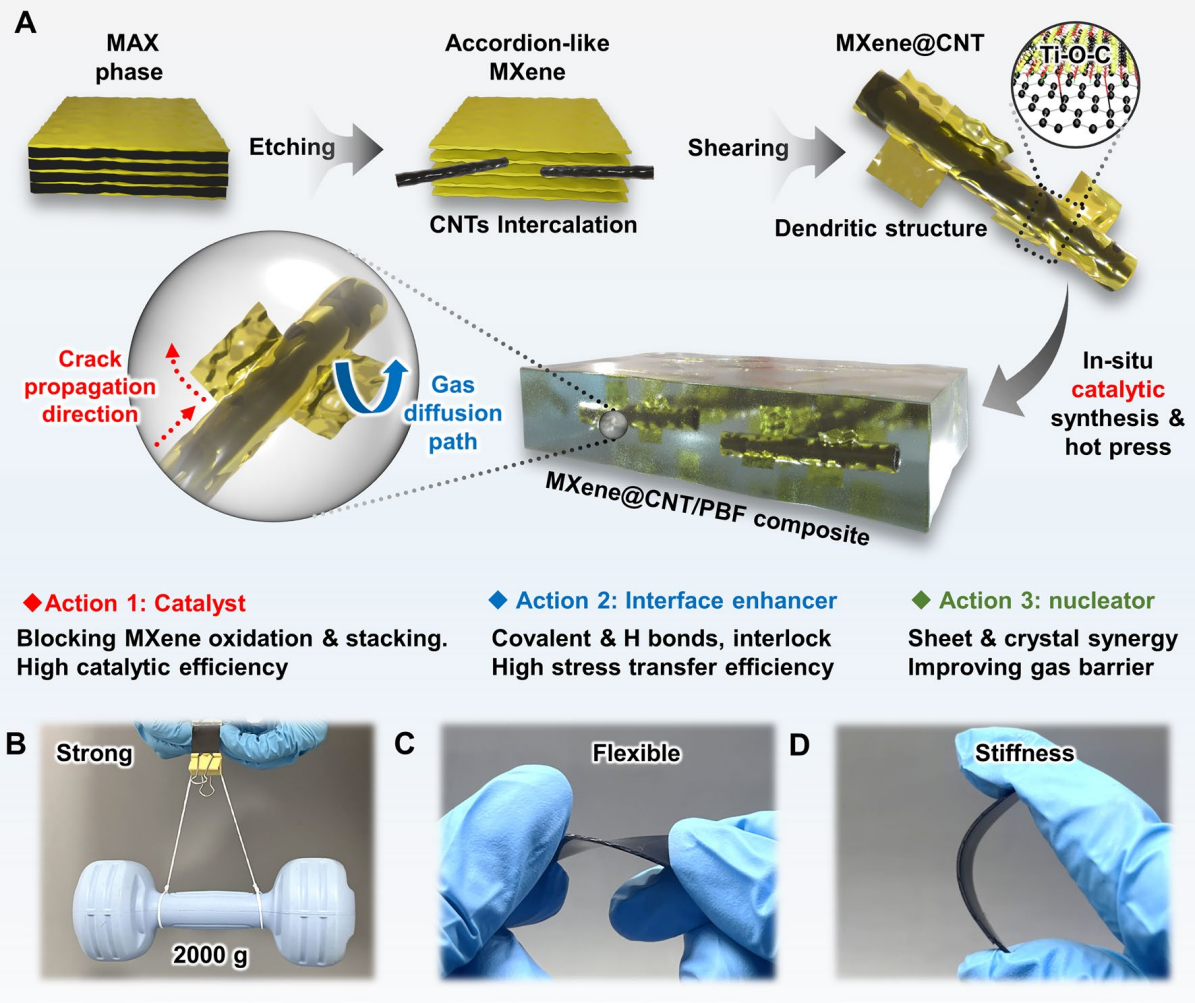
Conceptual design, constructing process of MXene@CNT heterostructure and MCP nanocomposite.** A** Scheme for fabricating the multi-scale dendritic MXene@CNT heterostructure via a facile intercalation exfoliation combines high-speed stirring, and the MCP polyester nanocomposites via in situ catalytic polymerization strategy. The dendritic MXene@CNT heterostructure can achieve the multi-scale dissipation of stress and multi-dimensional barrier for gas medium. Digital photograph of MCP polyester nanocomposite, the superior **B** mechanical strength, **C** toughness, and **D** stiffness can make it withstand 2000 times its own weight, twisting and folding, and no damages are observed

Recently, Wu et al. [29] found that curled 2D MXene into 1D fiber could effectively address the problems of agglomeration and re-stacking of MXene nanosheets. Surprisingly, it is a wonder that this behavior characteristics can be combined with the 1D CNT fiber. There is thus a great interest to develop novel hetero-structured MXene@CNT filler, which utilize the 2D MXene nanosheets to wrap 1D CNT fiber for constructing a multi-scale dendritic structure (Fig. 1A), which not only can achieve the synergistical dispersion of the two, but also inhibit the MXene oxidation through forming Ti–O–C covalent bonds with residual hydroxyl groups in CNT. Such dendritic hetero-structured MXene@CNT are envisaged to enhance catalytic and nucleation efficiency of MXene@CNT by exposing more active sites and endow nanosheet/crystal dual barrier effects. Meanwhile, it can form multi-scale interface interactions (i.e., chemical bonds and mechanical interlocks) with matrix to realize high stress transfer and thus enhance mechanical properties. To demonstrate this hypothesis, here we design and construct the multi-scale dendritic MXene@CNT heterojunction with superior dispersion and structure stability, via wrapping the 2D MXene nanosheets onto 1D CNT fiber surfaces. In view of the high adjustability and crystallinity of molecule chains of bio-based PBF polyester. Then, the hetero-structured MXene@CNT was in situ added into PBF matrix to fabricate MXene@CNT/PBF (denoted as MCP) polyester nanocomposites. Based on multi-inlaid MXene nanosheets on the surface of dendritic structure, the MXene@CNT can act triple roles, i.e., polycondensation catalyst, crystal nucleator, and interface enhancer of PBF. Benefiting from the distinctive multi-scale energy dissipated (MSED) structure, this MCP nanocomposite has exceptional tensile strength (≈101 MPa), stiffness (≈3.1 GPa), and toughness (≈130 MJ m^−3^); low gas permeability coefficient (e.g., O_2_ 0.0187 barrer, CO_2_ 0.0264 barrer, and H_2_O 1.57 × 10^−14^ g cm cm^−2^ s Pa); superior reprocessability, UV resistance, and solvent resistance, making it a suitable substitute for petrochemical-based plastics. Significantly, our work provides a novel design concept to construct sustainable high-performance materials based on catalysis-interfacial strengthening integration strategy.

## Experimental Section

### Materials

Ti_3_AlC_2_ (MAX phase) powder was provided Ningbo Beijiaer New Material Co., Ltd (Beijing, China). Carbon nanotube (CNT, length 15 μm, ≥ 97%) powder was purchased from Turing Evolution Technology Co., Ltd. (Shenzhen, China). Lithium fluoride (LiF, ≥ 99.99%), 1,4-butanediol (BDO, 99%), tetrabutyl titanate (TBT, 99%), methanol (MeOH, AR), ethanol (EtOH, AR), dimethyl sulfoxide (DMSO, AR), acetone (AC, AR), N,N-dimethylformamide (DMF, AR), tetrahydrofuran (THF, AR), sodium hydroxide (NaOH, AR), hydrochloric acid (HCl, 35 wt%), sulfuric acid (H_2_SO_4_, 98 wt%), triphenyl phosphate (TTP) were purchased from Aladdin Reagent Co. Ltd. (Shanghai, China). 2,5-Furandicarboxylic acid (FDCA, 99.9%) monomer was purchased from Ningbo Jisu New Material Technology Co., Ltd. (Ningbo, China). All chemical regents and materials were used without further purification.

### Preparation of Dendritic Hetero-Structured MXene@CNT

MXene@CNT was prepared by acid etching combined shear mixing [14, 30]. MAX phase powder was added to 50 mL HCl (9 M) solution with 5 g of LiF, and the mixture was stirred for 24 h at 35 °C. Afterward, the product was filtered and washed (25 mL × 5) with distilled water (DIW) to obtain accordion-like multi-layered MXene precursor. Then, a DIW solution containing multi-layered MXene (500 mg) and CNT (250 mg) was sonicated for 5 min. Next, the mixed solution was shear treated for 8 h at 3000 rpm until reaction between the MXene nanosheets and CNT was completed. The as-obtained dispersion of MXene@CNT was kept standing for 5 h. After removing the solid residues at the bottom, the hetero-structured MXene@CNT composites were obtained by vacuum-assisted filtration and freeze dried. To quantify the conversion efficiency of the MXene@CNT, the productivity was calculated by the weight of the MXene@CNT remaining in the upper liquid.

### Synthesis of Dimethyl Furan-2,5-Dicarboxylate (DMFD)

The DMFD monomer was first synthesized through esterification reaction between FDCA and MeOH. In a typical experiment, 0.5 mol of FDCA and 10 mol of MeOH were added into a three-necked flask (1000 mL) equipped with mechanical stirrer, and then, 2 mL of H_2_SO_4_ catalyst was added. The system was stirred and refluxed at 90 °C for 3 ~ 5 h at 150 rpm. In this process, the excessive MeOH was distilled, and the generated dimethyl ester was precipitated as white powder after cooling to room temperature. The precipitated was collected through filtration and washed with DIW for several times. Then, the DMFD crystal was obtained after drying at 120 °C in the vacuum oven and purified by sublimating.

### Synthesis of MCP Nanocomposites

MCPs were synthesized through in situ esterification and polycondensation of DMFD monomer, BDO monomer, and MXene@CNT (as catalyst and functional filler). Prior to the reaction, a certain amount of MXene@CNT powder (the mass fraction of filler was 0.1, 0.2, and 0.3 wt% of DMFD monomer) was evenly dispersed in BDO by stirring for 24 h at room temperature. The molar ratio of BDO to DMFD was set into 1.6. In the initial stage, a three-necked round-bottom flask (3000 mL) equipped with a mechanical stirrer, N_2_ inlet, and reflux condenser was filled with DMFD, BDO, and MXene@CNT. Before heating treatment, the mixed system was purged for three times using high-purity N_2_ to ensure an inert atmosphere. The esterification reaction occurred at 180 °C for 3 ~ 5 h under an N_2_ atmosphere until about 95% of the theoretical amount of MeOH had been distilled out. During the polycondensation reaction step, the temperature was adjusted to 220 °C and the pressure was reduced to about 15 Pa. The reaction continued for 4 ~ 6 h until the torque value of the stirrer remained constant, suggesting that the reaction had reached its endpoint. Finally, atmospheric pressure was restored through the N_2_ inlet to obtain the desired product. According to the faction of fillers, the nanocomposite specimens were denoted as MCP1 (0.1 wt%), MCP2 (0.2 wt%), and MCP3 (0.3 wt%), respectively. For comparison purposes, the virgin PBF samples were also synthesized by using TBT and MXene as catalyst, respectively. The synthesis conditions for virgin PBF catalyzed by TBT and MXene were identical with MCPs, and the contents of TBT were 1 mol‰ while the MXene was 300 ppm based on DMFD, respectively.

### Characterizations

The detailed characterization approaches can be found in Supporting Information.

## Results and Discussion

### Design of Dendritic MXene@CNT and MCPs

As typical carbon nanomaterials, 1D CNT and 2D MXene have drawn great science interest due to their outstanding mechanical and chemical properties [16, 23]. However, the inert nature of CNT often causes poor dispersion and interface issues [17], which significantly affects its performance in functional polyester nanocomposites fillers. Due to the strong interlayer interactions (e.g., hydrogen bonds, electrostatic interactions, and van der Waals forces) between individuals and oxygen sensitivity, MXene nanosheets tend to agglomeration and oxidative deterioration [25, 27], which inevitably decreases their exposed surface areas and catalytic efficiency for polyester polycondensation reaction. Many researches reveal that nanoscale heterostructures with multi-scale bonded junctions have improving performance in three dimensions, enabling them attractive for various prospective applications [28, 31, 32]. On the basis of this consensus, integrating 1D CNT and 2D MXene can be a promising approach to construct multi-scale structures with complementary properties (e.g., large specific surface and high load transfer along both 1D axes and 2D planes). One commonly method is simply mixing CNT and MXene to obtain hybrids [33, 34]; however, the weak couple-interface structures between the two limit their performance.

To realize the uniform dispersion, strong interaction, and optimal properties of CNT and MXene in polyester matrix, we construct a dendritic hetero-structured MXene@CNT by a facile shear mixing strategy (Fig. 1A). According to Dong et al. [35] by utilizing the electrostatic attraction between the negatively charged CNT and positively charged edges of MXene, MXene nanosheets could be easily exfoliated by the intercalated CNT under high-speed shear force. Based on this, we introduce the high-speed shear forces in the CNT and accordion-like multi-layered MXene (Fig. S1) exfoliation system, to induce the self-assembly and multi-scale wrapping of 2D MXene on 1D CNT’s surface to obtain dendritic MXene@CNT hybrids. The MXene@CNT junctions are dominated by Ti-O-C covalent bonds, effectively inhibiting the oxidation of MXene. More importantly, the multi-scale dendritic structure successfully addresses the agglomeration problem of MXene and achieves the synergistically high dispersion of 1D nanotubes and 2D nanosheets. Subsequently, the dendritic hetero-structured MXene@CNT is then used as polycondensation catalyst and functional filler (interfacial modifier) of bio-based PBF to in situ synthesize MCP nanocomposites. Because the multi-scale embedded MXene nanosheets on the dendritic MXene@CNT can expose more surface areas, the catalytic polycondensation reaction of PBF polyester has been successfully realized. More significantly, the dendritic MXene@CNT can form multiple interface interactions with PBF matrix through mechanical interlocking and chemical bonding (i.e., covalent and hydrogen bonds) to obtain high stress transfer efficiency and thus significantly enhance the mechanical strength (Fig. 1B), toughness (Fig. 1C), and stiffness (Fig. 1D) of MCP. Moreover, uniformly dispersed MXene@CNT hybrids can serve as high-efficient nucleator, under the nanosheet/crystal dual physical barrier effects, to greatly improve the gas barrier properties of MCP nanocomposites.

### Construction and Characterization of Dendritic MXene@CNT

**Fig. 2:**
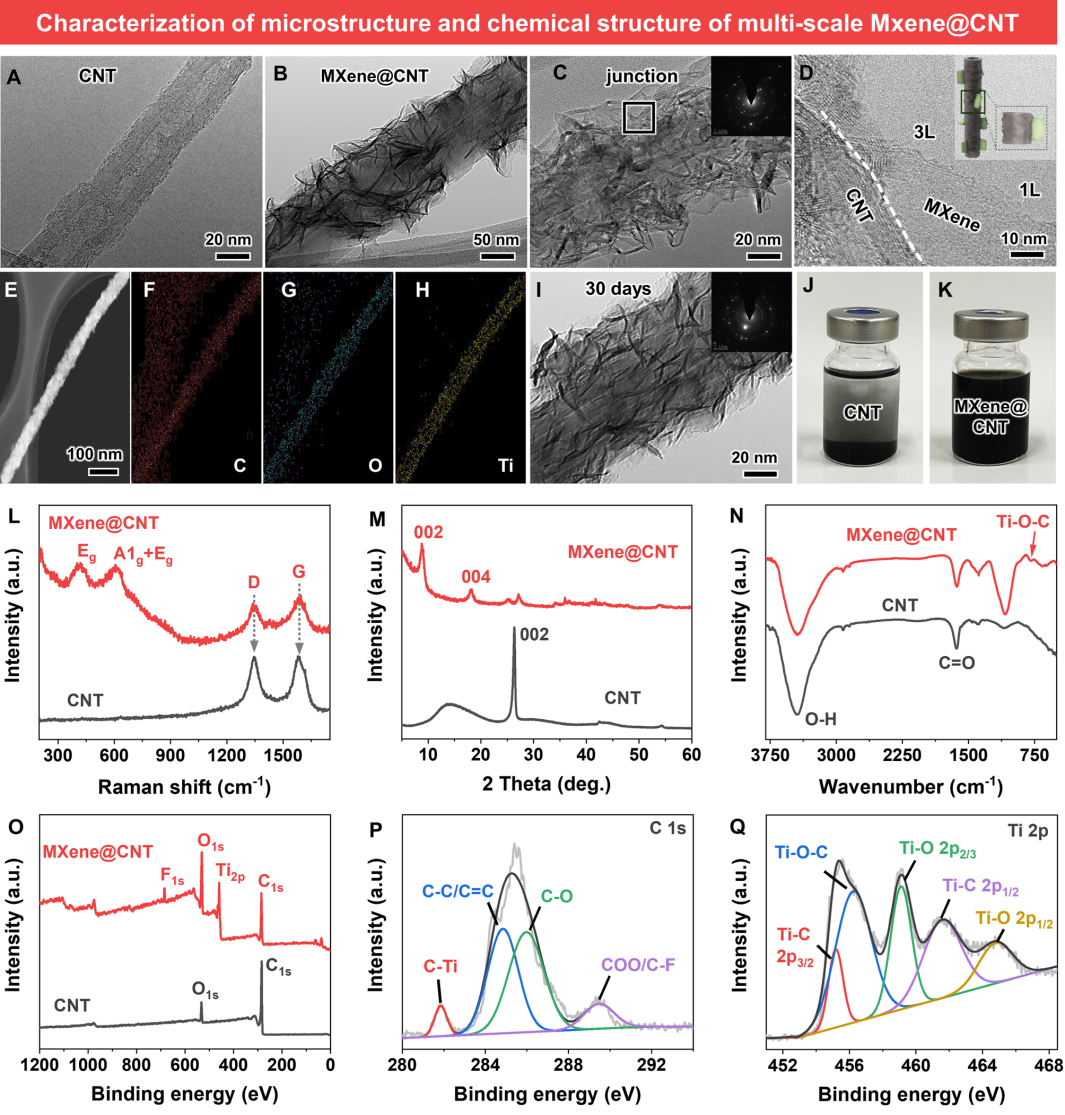
Characterization of microstructure and chemical structure. TEM image of **A** CNT and **B** MXene@CNT. **C** High-resolution TEM image of MXene@CNT. **D** The junction structure of MXene@CNT. **E–H** Surface and corresponding EDS mapping images for C, O and Ti. **I** TEM image of MXene@CNT after storage for 1 week. **J** and **K** Digital photographs of CNT and MXene@CNT BDO dispersions. **L** Raman spectra, **M** XRD results, and **N** FTIR, spectra of CNT and MXene@CNT. **O** XPS wide spectra, **P** C_1s_ fine, and **Q** Ti_2p_ fine spectra of CNT and MXene@CNT

SEM and TEM were used to verify the microstructure of dendritic hetero-structured MXene@CNT. Compared with unmodified CNT (Fig. 2A), the as-obtained MXene@CNT shows multi-scale dendritic structure (Figs. 2B and S2), in which 2D MXene nanosheets are uniformly self-assembled onto 1D CNT fibers through surface wrapping. Besides, due to the multi-scale wrapping of MXene nanosheets on CNT‘s surface, the diameter increases from ≈30 nm for virgin CNT to ≈60 nm for MXene@CNT. We further performed high-resolution TEM (HRTEM) to study lattice registration at the nanosheet/nanotube interfaces (Fig. 2C). The nanosheets, nanotubes, and nanosheet/nanotube interfaces illustrate lattice spacing of 0.26 and 0.32 nm (Fig. S3), which index to the (100) plane of MXene and (010) axe of CNT, respectively [33, 35]. From Fig. 2D, the single- and few-layered MXene nanosheets in the MXene@CNT hybrids are closely wrapped on CNT. EDS mapping images (Fig. 2E-H) obtained from these HRTEM images reveal the uniform distribution of MXene nanosheets in the dendritic hetero-structured MXene@CNT. As shown in Fig. 2I, HRTEM image indicates that the multi-scale structure successfully reduces the oxidation sensitivity of MXene nanosheets, in which the surface wrapped MXene remained its intact lattice structure after being stored for 30 days under ambient conditions.

The agglomeration issue of fillers in polymeric matrix is a key scientific and technological challenge, the solution to which will enhance the utilization efficiency of fillers and lead to increased performance for nanocomposites. To study the stability of MXene@CNT, the dispersions of CNT and MXene@CNT in BDO with a concentration of 3 mg mL^−1^ were monitored after mild stirring. In Fig. 2J, K, the CNT dispersion shows complete aggregation and deposition, while the MXene@CNT dispersion has superlative dispersion after 1 week of sedimentation. This reveals that the MXene-modified CNT has high dispersibility, which is well agreed with the previously reported result by Wu et al. [29]; they confirmed that the curled MXene could avoid the aggregation of nanosheets. In conclusion, MXene@CNT was constructed from MXene nanosheets together with CNT through shear mixing, in which MXene was simultaneously restricted the settlement behaviors of nanosheets and nanotubes.

Furthermore, molecular dynamics (MD) simulations were performed to reveal how MXene nanosheets modulated the CNT‘s surface, and how MXene redounded the synergistic dispersion of CNT. In Fig. S4, the CNT keeps stable while being blended and wrapped by MXene nanosheets. After CNT was completely wrapped by MXene, the superfluous nanosheets were partly stacked into single- or/and multi-layered MXene with outstretched edges, forming a heterostructure in equilibrium [9, 10, 30]. There is no obvious energy barrier is detected in the interaction energy profile (Fig. S4A) of CNT with the decreasing of the distance between two nanotubes, revealing that the aggregation of CNT is energetically favorable. In the MXene@CNT system (Fig. S4B), the interaction energy abruptly augments at a certain position and gets the maximum of 32.89 eV (Table S1). The great energy barrier is caused by the coexisting heterostructure of 1D CNT and 2D MXene through the acute deformation o nanosheet during the aggregation and re-stacking process [36, 37]. MXene and CNT migrate and morph as well as concurrently interlock each other as a unit [30], in the exploring equilibrium process. Armed with theoretical and experimental analysis, the results uncover that multi-scale MXene-tailored CNT effectually increase the interaction energy barrier, enabling aggregation and re-stacking difficult, and thus greatly enhancing the dispersed ability.

In Fig. 2L, the typical Raman D-band peak (~ 1350 cm^−1^) and G-band peak (~ 1580 cm^−1^) are detected in both CNT and MXene@CNT, which are related to the disorder degree of structure and the *sp*^2^ hybridization level of graphite carbon atoms [33, 34]. The Raman spectrum from the hetero-structured MXene@CNT shows that the wrapped MXene nanosheets have the typical A_1g_ symmetry out-of-plane vibrations of Ti and C atoms at ~ 720 cm^−1^, and the in-plane E_g_ vibrations of Ti, C atoms, and terminal functional groups at 284 and 625 cm^−1^. These characteristic peaks are expected for the exfoliation of MXene nanosheets. Besides, due to the inclusions in CNT and the crumpled and stacked MXene nanosheets, the coexistence of CNT and MXene induced Raman peak widening [30]. The I_D_/I_G_ ratio of the MXene@CNT increases to  ~ 1.1, from that of ~ 0.9 for virgin CNT, which is ascribed to the possible reaction between MXene and CNT [38].

XRD was applied to further study the heterostructure of MXene@CNT. As illustrated in Fig. 2M, compared with the trenchant diffraction (002) peak at ~ 26.55° for virgin CNT, the MXene@CNT appears a decreased and wide peak, suggesting the increased interlayer *d*-spacing of CNT. This is attributed to the effective MXene nanosheets wrapping and the possible formation of interface bonding interactions e.g., covalent bonds and hydrogen bonds among MXene@CNT hybrids [14, 39]. In addition, MXene has a characteristic 2θ peak for CuKα radiation at ~ 6.52°, which indicates that multi-layered MXene have been successfully exfoliated into few-layered nanosheets.

**Fig. 3:**
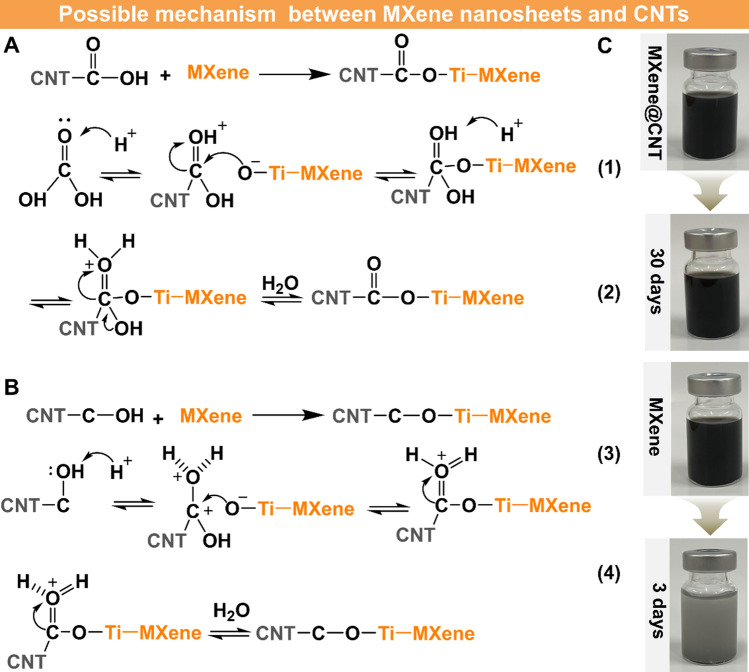
Possible mechanism of formation of Ti–O–C covalent bonding between MXene nanosheets and CNT. **A** The residual carboxyl group (-COOH) of CNT reacts with MXene nanosheets to form Ti–O-C covalent bonding via nucleophilic substitution and dehydration reaction of Eqs. (1) and (2). **B** The residual hydroxyl group (–OH) of CNT reacts with MXene nanosheets through nucleophilic substitution and dehydration reaction, producing interfacial Ti–O–C covalent bonding of Eqs. (3). **C** Digital photos of MXene@CNT and MXene for storage in different times to revealing their stability

Figure 2N shows FTIR results of CNT and MXene@CNT; a broad absorption peak at ~ 3300 cm^−1^ for the hydroxyl groups (−OH) and a sharp absorption peak around at ~ 1720 cm^−1^ for the carboxyl groups (−COOH) are be found for CNT [9, 34]. As expected, these two peaks are also detected in the MXene@CNT, owing to the coexistence of CNT and MXene nanosheets. Furthermore, a new absorption peak at ~ 840 cm^−1^ is found, which is assigned to the Ti–O–C [14, 27]. Results confirm the formation of Ti–O–C bonds between CNT and MXene, which is direct evidence for in the oxidation stability of MXene nanosheets in the hetero-structured MXene@CNT system. XPS (Fig. 2O) illustrates the appearance of Ti and F elements, revealing that MXene nanosheets have successfully reacted with CNT. As shown in Fig. S5, the C_1s_ spectra of the virgin CNT display three characteristic peaks at binding energies of 284.8, 288.3, and 290.6 eV, which are assigned to the graphite-phase aromatic rings of C–C/C=C and residual –OH and –COOH functional groups of C–O and O=C–O of, respectively [22]. In contrast, the C_1s_ spectra (Fig. 2P) of the MXene@CNT exhibit the atomic percentage of C–O increases; a new peak that is assigned to Ti–O–C at the binding energy of 211.9 eV is found. Furthermore, the Ti_2p_ spectra (Fig. 2Q,) of the MXene@CNT have five typical peaks at binding energies of 455.0, 456.5, 458.1, 461.0, and 463.4 eV, which belong to the bonds of Ti–C_2p3/2_, Ti–O–C, Ti–O_2p3/2_, Ti–C_2p1/2_, and Ti–O_2p1/2_ [40], respectively. All these results demonstrate the formation of Ti–O–C covalent bond between CNT and MXene nanosheets (detail see Fig. 3), which is well agreed with the Raman and FTIR results [41].

### In Situ Catalytic Synthesis and Structural Characterization of MCPs

**Fig. 4:**
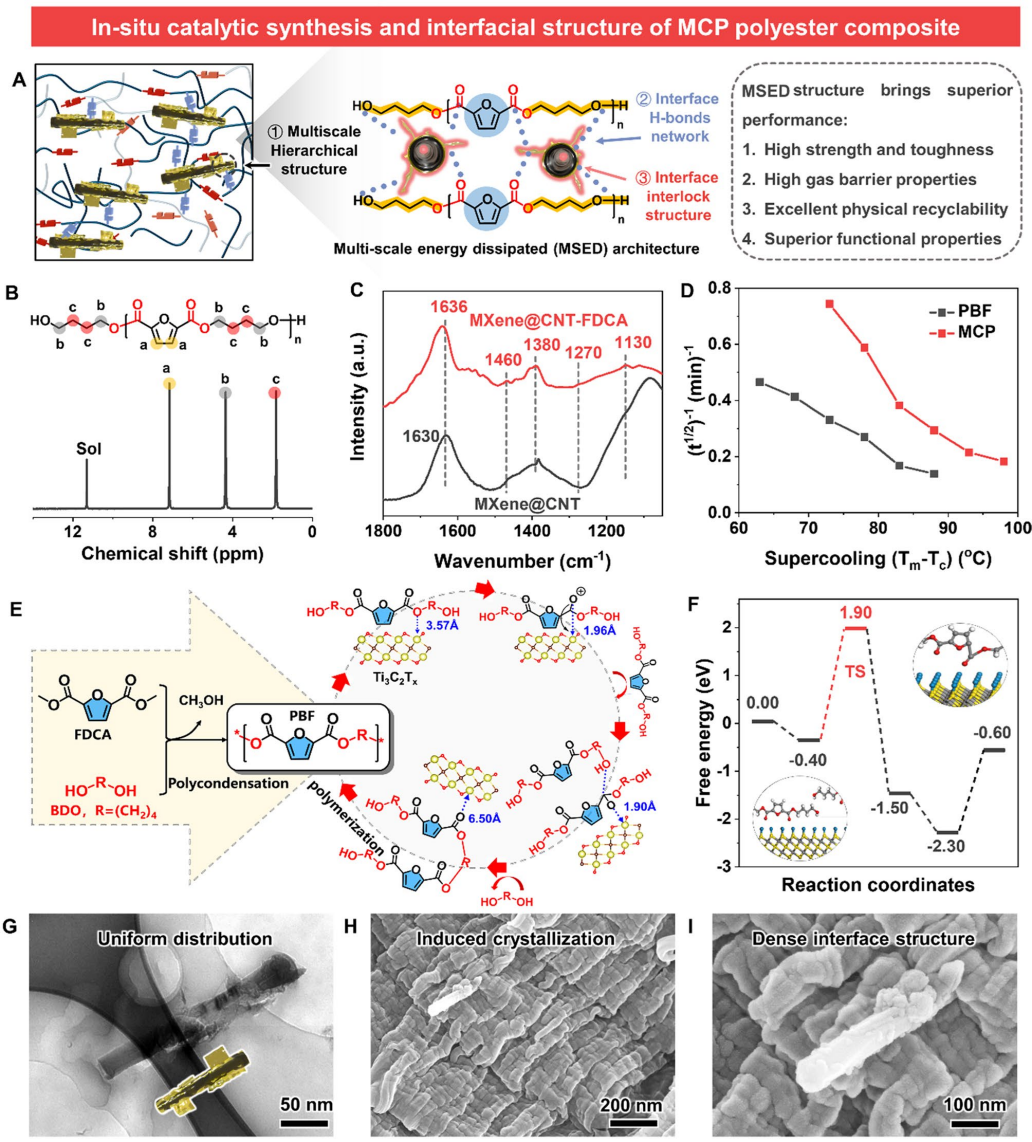
In situ catalytic polymerization and microstructures.** A** Schematic illustration of the structure of MCP polyester nanocomposite with a hierarchical energy dissipated architecture, and the corresponding superior performance endowed by it. **B** ^1^H NMR of MCP sample. **C** FTIR spectra of MXene@CNT and MXene@CNT-FDCA. **D** Isothermal crystallization half-time as function of crystallization temperature. **E** Reaction pathway and **F** important structures of PBF catalyzed by MXene. **G** Representative cross-sectional TEM image of MCP nanocomposite. **H** low- and **I** high-resolution cross-sectional TEM image of MCP nanocomposite

To overcome the performance contradiction of FDCA-based polyesters, we try to design a multi-scale energy dissipated (MSED) architecture in PBF matrix (Fig. 4A). The dendritic MXene@CNT can in situ entangle with PBF chain segments by interfacial chemical bonding (i.e., covalent and hydrogen bonds) and mechanical interlocking, enhancing synergistically stress transfer, and thus achieving the overall performance upgrades of MCP materials. Inspired by the catalytic acidity stemmed from abundant surface functional groups, we utilize MXene as a polycondensation catalyst for achieve the successful synthesis of PBF and MCP polyester nanocomposites. More importantly, MXene is green and safe without heavy metal composition. The MXene is enriched with a multitude functional groups (see Fig. 2P, Q), endowing it a Lewis − Bronsted acidic catalyst [42]. To study the acidity information, pyridine FTIR of MXene was carried out (Fig. S6A). Bands illustrated at 1450 and 1600 cm^−1^ can be assigned to the pyridine anchored on strong Lewis acid sites, while the bands at 1485, 1540, and 1575 cm^−1^ belong to strong Lewis/Brönsted acid sites, Brönsted acid sites, and weak Lewis acid sites, respectively [43, 44]. In Fig. S6B, the model shows that the strong acidity mainly steamed from the electron-withdrawing effect of − F atoms in MXene, in which the Lewis acid sites are ascribed to the Ti atom layer, while the Brönsted acid sites are ascribed to the −OH groups. Thus, MXene is a homogeneous catalyst, which is expected to have a superior catalytic activity for PBF polycondensation synthesis.

The ^1^H NMR spectra of PBF specimens catalyzed by multi-layered MXene (Fig. S6D) and MXene@CNT (Fig. 4B) are well agreed with the one catalyzed by TBT (Fig. S6C), revealing that the successful synthesis of PBF and MCPs, and the small addition of nanoadditives did not affect the molecular structures of polyesters. Additionally, the intrinsic viscosities of MCPs are higher than that of TBT-catalyzed PBF (Table S2), which suggests a higher catalytic efficiency of MXene compared to TBT. To confirm that MXene@CNT can in situ bridge PBF chain segments via forming covalent bonds and hydrogen bonds, FDCA monomer was selected to react with MXene@CNT. FTIR spectrum (Fig. 4C) of the resultant MXene@CNT-FDCA shows a conversion of hydroxyl groups to ester bonds (around at ~ 1630 cm^−1^) and hydrogen bonds (~ 1460 cm^−1^) [45, 46], demonstrating that MXene@CNT filler can as a special monomer to involve the polymerization reaction of PBF. Significantly, based on the fiber-like morphology of dendritic MXene@CNT, it has the potential function to be a crystal nucleator within the PBF matrix. As shown in Fig. 4D, the shortest t^1/2^ (half-time of crystallization, ~ 72 s) of MCP, whereas the virgin PBF is ~ 132 s (Fig. S7). Results are agreed with previous works [24, 25], suggesting that dendritic MXene@CNT as a heterogeneous nucleation agent can promote the crystallization, reorganization, and packing of PBF chains. Besides, the crystallinity degree increases from 19.8% for PBF to 23.2% for MCP3 (Fig. S8).

To investigate the reaction mechanism and the role of MXene in the polycondensation of PBF, we carried out DFT calculations. Figure 4E shows the presumptive mechanism of PBF polycondensation catalyzed by MXene. The results reveal the minimum-energy pathway for the polycondensation of PBF catalyzed by using MXene, suggesting its superior catalytic capability and the typical Lewis acid catalytic mechanism [24, 42]. In Fig. 4F, in the transition state (TS), the distance of *d*_O–Ti_ (the O atoms of the carbonyl groups and Ti layers in MXene) decreases, and the O atoms of the carbonyl groups interconnect with Ti in MXene [25, 43, 44]. As a result, the electron cloud density of the C atom in the carbonyl group reduces, which enhance the positive charge of carbonyl carbon by inducing electron transfer of carbonyl groups, facilitating attacks of lone pair electrons on hydroxyl groups, and thus leading to the growth of PBF molecular chains.

Subsequently, all polyester films were fabricated through the same optimal process parameters by a hot-pressing strategy. Due to the same fabrication process, the interfacial morphologies of PBF (Fig. S9A) and MCP (Fig. 4G) films are almost identical, which further confirms that the addition of MXene@CNT did not disturb the polymerization of polyesters. The cross-sectional TEM images (Fig. S9B, C) further show that MXene@CNT is well dispersed in PBF matrix, without any aggregation or bundle structure. As shown in Fig. 4H, I, the dendritic hetero-structured MXene@CNT is tightly embedded in the PBF matrix, forming a strong interfacial bond structure. Moreover, the crystallinity degree of MCPs (Fig. S9E, F) increases significantly compared with virgin PBF (Fig. S9D). Therefore, benefiting from the advantageous structure of dendritic MXene@CNT heterojunction, the small addition of it can achieve the important structural optimization for polyester nanocomposites.

### Mechanical Properties and Enhancement Mechanism

**Fig. 5:**
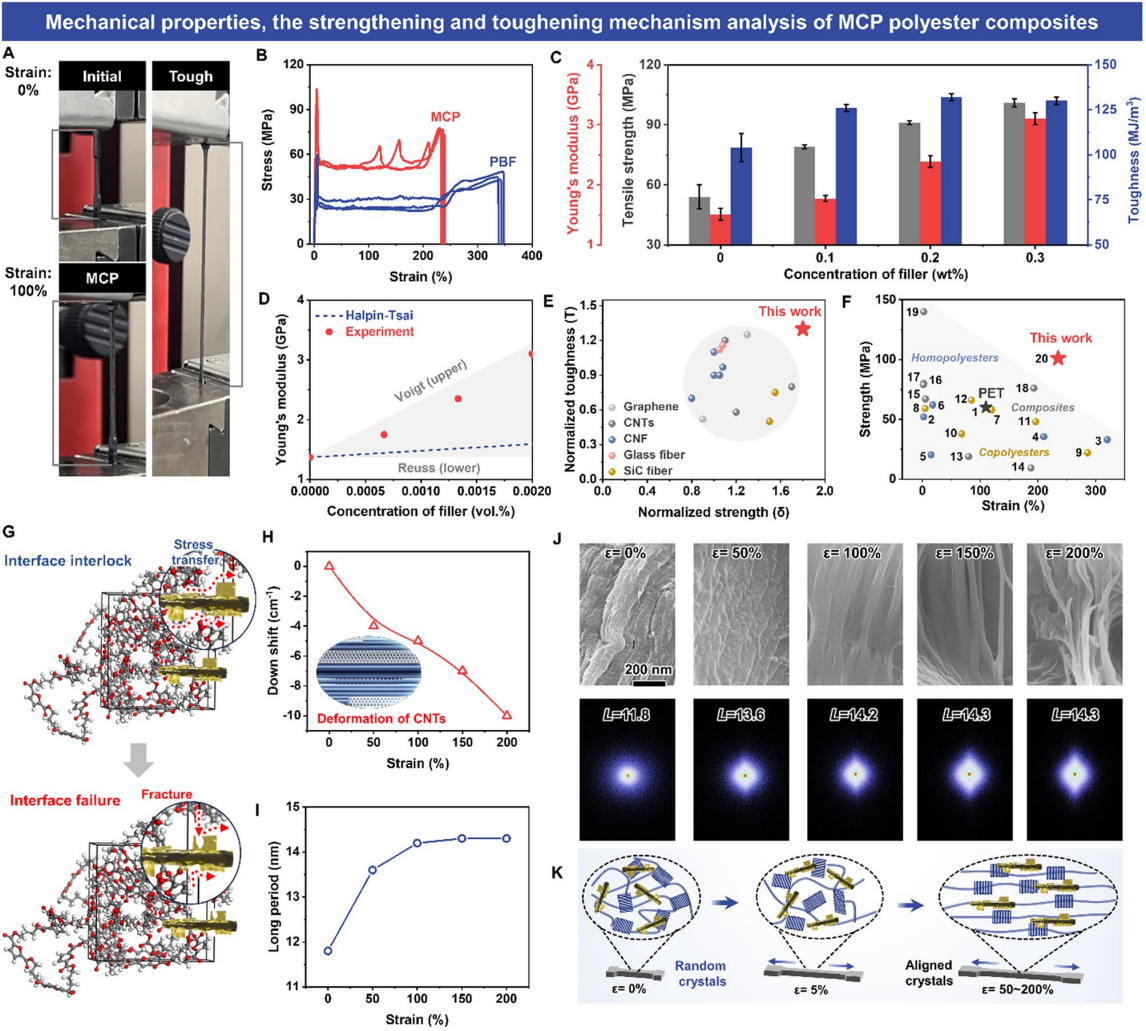
Mechanical properties and corresponding mechanisms. **A** Digital photographs of typical stretching process for MCP. **B** Typical stress-strain plots of PBF and MCP films. **C** Calculated strength, modulus, and toughness of PBF and MCP nanocomposite with different filler contents. **D** Comparison of theoretical and experimental values of modulus for MCP films. **D** Comparison of theoretical and experimental values of modulus for MCP films. **E** Strengthening and toughening effects of MXene@CNT and other-type nano-fillers. **F** Comparison of mechanical properties of MCP films with other FDCA-based materials. The references associated with the sample numbers in this curve are in Table S4. **G** Scheme for the stress transfer pathways and failure process of MXene@CNT in the PBF matrix. **H** The strain dependence of the G band downshifts in Raman frequency MCP films. **I** Long period extracted from SAXS patterns as function of strain.** J** SEM images, 2D SAXS patterns, and the changes of crystals and lamellae during stretching for MCP films

As covalent and hydrogen bonds can induce densification and strengthen the multi-scale interfacial interactions, our MCP nanocomposite films have significantly improved mechanical strength, stiffness, and toughness (Fig. 5A). The tensile stress–strain curves of virgin PBF and MCP films are shown in Figs. 5B and S10. The virgin PBF films have a tensile strength of 54 ± 6 MPa, a Young’s modulus of 1.5 ± 0.1 GPa, and a toughness of 104 ± 5 MJ m^−3^ (Fig. 5C and Table S3). After embedding MXene@CNT, the tensile strength, Young’s modulus, and toughness of MCP films greatly increase. The mass ratio of MXene@CNT significantly influences the mechanical properties of the MCP films. As increasing filler content, the tensile strength and Young’s modulus of MCP films increase (Fig. 5C), but the tensile strain reduces (Fig. S10). This is ascribed to the fact that more fillers and crystals as crosslinking points to improve crosslinking density in MCP films, whereas the inhibition of polyester chain mobility cause reduced elongation at break [47]. Specifically, when the content of MXene@CNT is 0.3 wt%, the optimized MCP3 films show the highest tensile strength of 101 ± 2 MPa and toughness of 130 ± 2 MJ m^−3^ with the Young’s modulus of 3.1 ± 0.1 GPa; the strength and toughness are 1.87 and 1.25 times higher than virgin PBF films. This exceptional strength and toughness are attributed to the interfacial synergetic interactions of dendritic hetero-structured MXene@CNT and PBF molecules.

To verify the reliability of mechanical properties enhancement, we introduce the theoretical models of the nanocomposites based on the *Voigt* approximation model (superior limit), the *Reuss* approximation model (lower limit), and the *Halpin–Tsai* model (empirical model) [9, 17, 30].

1 $$\text{Voigt approximation model}:{E}_{MCP}=v{E}_{MXene@CNT}+\left(1-v\right){E}_{PBF}$$

2 $$\text{Reuss approximation model}: \frac{1}{{E}_{MCP}}=\frac{v}{{E}_{MXene@CNT}}+\frac{1-v}{{E}_{PBF}}$$

*Halpin–Tsai* theoretical model:

3 $$\begin{gathered} E_{MCP} = E_{PBF} \left[ {\frac{3}{8} \cdot \frac{{1 + \eta_{1} \left( {2l_{MXene@CNT} /d_{MXene@CNT} } \right)v}}{{1 - \eta_{1} v}} + \frac{5}{8} \cdot \frac{{1 + 2\eta_{2} v}}{{1 - \eta_{2} v}}} \right] \hfill \\ \eta_{1} = \frac{{E_{MXene@CNT} /E_{PBF} - 1}}{{E_{MXene@CNT} /E_{PBF} + 2l_{MXene@CNT} /d_{MXene@CNT} }} \hfill \\ \eta_{2} = \frac{{E_{MXene@CNT} /E_{PBF} - 1}}{{E_{MXene@CNT} /E_{PBF} + 2}} \hfill \\ \end{gathered}$$

where *E*_PBF_, *E*_MCP_, and *E*_MXene@CNT_ are the modulus values of PBF films, MCP films, and MXene@CNT, severally. *v*, *l*_MXene@CNT_, and *d*_MXene@CNT_ are the volume fraction, length, and diameter of the MXene@CNT. The modulus of hetero-structured MXene@CNT is approximated as that of CNT (950 GPa). In Fig. 5D, the modulus of MCP films is much higher than the *Halpin–Tsai* theoretical predicted values, and very close to the *Voigt* upper limit, revealing the validity of our experimental results. As shown in Fig. 5E, our MXene@CNT-reinforced polyester nanocomposites possess a superior combination of strength and toughness compared with the previously reported nanocomposites containing different fillers, e.g., CNT, graphene, carbon nanofibers (CNFs), glass fibers, and SiC fibers [9, 18–20, 30]. Furthermore, the mechanical properties of our MCP films are compared with and petrochemical-based PET [9], and renewable FDCA-based polyester materials such as homopolyesters, copolyesters, and polyester nanocomposites (Fig. 5F, and Table S4). The results demonstrate that the performance of our MCP films shows superior tensile strength (≈101 MPa) and superior elongation at break (≈237%) than those of petrochemical-based PET (60 MPa and 110%). In addition, our MCP films not only have the best integration of strength and elongation at break, but also show a higher modulus (≈3.1GPa) than most FDCA-based polyester materials. Therefore, MCP holds great promise for applications in structural materials and engineering plastics.

As shown in Fig. 5G, the multi-scale propagating of cracks caused by the dendritic hetero-structured MXene@CNT is the main “*external toughening*” mechanism for the MCP films [10, 14, 15]. As a strong interfacial reinforcement, MXene greatly enhanced the bonding between MXene@CNT and polyester matrix. The MCP films show typical “island-like” fracture interfaces (Fig. S11), revealing pinning and propagating of cracks in the interfaces between MXene@CNT and matrix [30]. Additionally, the debonding and pullout of dendritic MXene@CNT from polyester dissipate more energy. All of these collectively jointly contribute to the improved strength and toughness of MCP. To further analyze the toughening mechanism of the resultant MCP films, in situ Raman spectroscopy was performed as shown in Figs. 5H and S11. The down-shift of G-band frequency of CNT in hetero-structured MXene@CNT was used to trail the stress transfer efficiency [10, 14]. Due to the integration of covalent and hydrogen bonds together in MCP films, the interfacial synergistic effect is realized; MXene@CNT shows continuous stress transfer during the whole strain of 0 to 200%, contributing to prevent the crack deflection. The G-band shifts constantly and reaches 10 cm^−1^ (Fig. 5H), leading to an persistent increase of the stress transfer efficiency [48]. Then, the tensile strength and toughness of MCP films are simultaneously improved.

Besides, the superior mechanical performance of MCP films was also benefited from its distinctive MSED structure. Because the MXene@CNT can act as crystal nucleator for PBF chains, which can create a large amount of phase-separation structures. These hard domains (crystal phase) can readily slide and dismantle to effectively consume energy via dynamic breaks [58] and to improve the mechanical properties. More importantly, fiber-like MXene@CNT can act as orientation templates for PBF chains during the stretching (Figs. 5I and S13), achieving strain-induced orientation of the amorphous phases (Fig. 4J) as well as create new fibrillar architectures to realize the “*internal toughening*” effect [9, 45–47]. Hence, these results from the experimental tests and mechanism analysis are generally consistent, suggesting that the synergy effects of “*external toughening*” and “*internal toughening*” of multi-scale structure caused by small incorporation of well-dispersed dendritic hetero-structured MXene@CNT can explain the effectively simultaneous enhancement in strength and toughness of MCP films.

### Reprocessability, Gas Barrier and Functional Performance

**Fig. 6:**
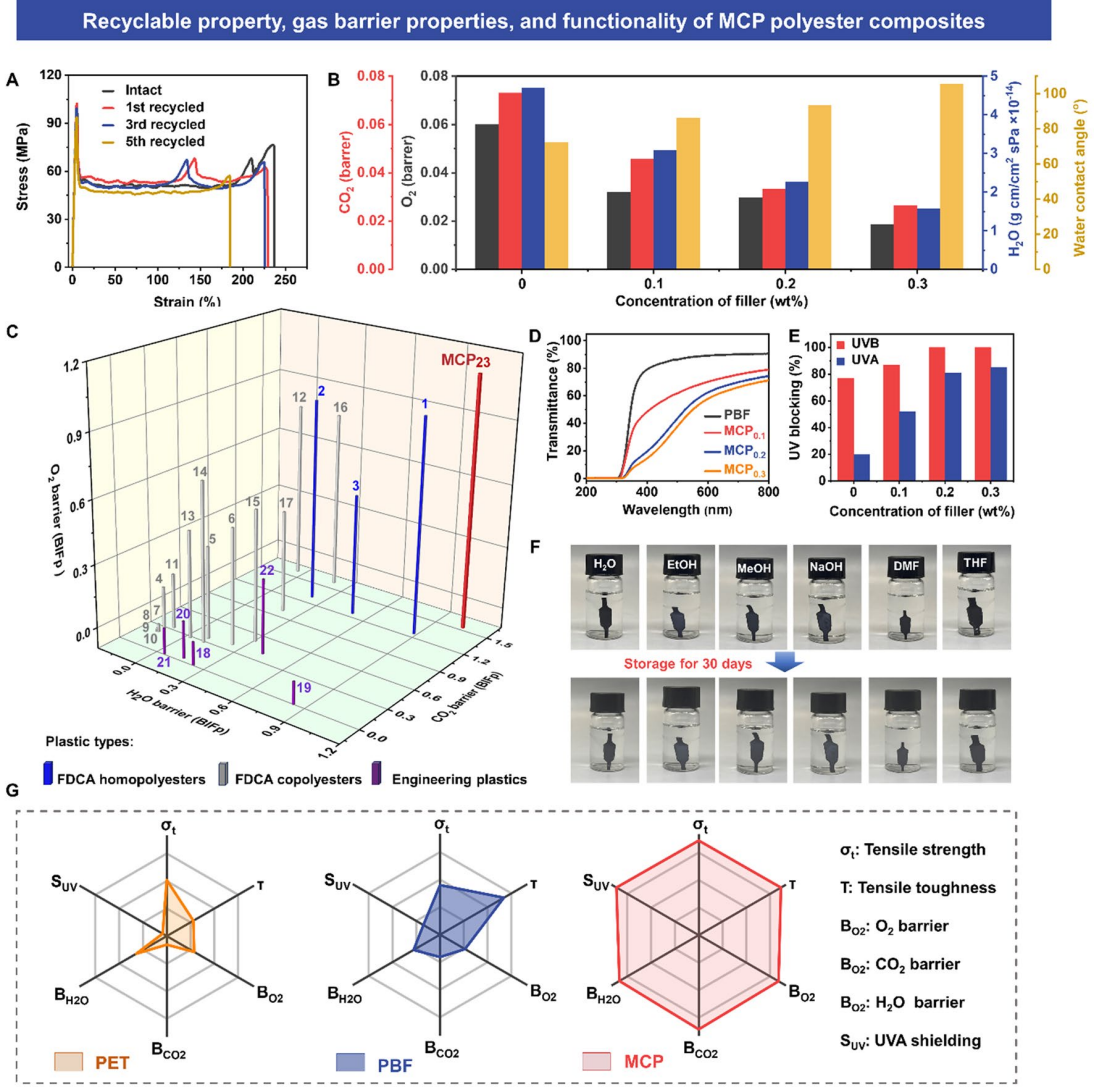
Recyclable, gas barrier and functionality. **A** Tensile strength-strain plots of physically recycled MCP films. **B** Calculated O_2_, CO_2_, H_2_O barrier properties and water contact angles of MCP films with different filler contents. **C** Comparison of O_2_, CO_2_, H_2_O barrier properties of MCP with other FDCA-based materials and Petro-based plastics. The references associated with the sample numbers in this curve are in Table S7. **D** UV–vis plots of MCP films with different filler contents and **E** corresponding UV-shielding properties. **F** Digital photographs of MCP films before and after immersion in different solvents for 30 days. **G** Radial plots comparing the tensile strength, tensile toughness, O_2_, CO_2_, H_2_O barrier, and UVA shielding properties for commercial PET, PBF, and MCP films

Physical recycling and reprocessing are important means to reduce the use of disposable plastics. Traditional polyester plastics cannot be well physically recycled due to the significant decrease in mechanical properties, causing serious resource waste. However, due to the synergy effects of “*external toughening*” and “*internal toughening*” in the MSED structure, our MCP films have expected reprocessability. Under hot pressing at 180 ~ 220 °C and 10 ~ 15 MPa for 10 ~ 15 min, new-generation MCP films can be easily prepared for achieving physical recycling. The physical reprocessing efficiency of MCP films was assessed through testing the mechanical performance (i.e., tensile strength and strain) of the reprocessed specimens (Fig. S14). The films show slightly decreases in tensile strength and toughness (elongation at break) after multiple recycling (Figs. 6A and S14), possibly owing to the decline in molecular weight during high-temperature reprocessing. Specially, the mechanical strength of the films was remained at a rate of up to  ~ 90% after five recycling (Table S5). This indicates that MCP films have superior physical recycling and reprocessing performance in practical applications due to the synergy effects in the MSED structure.

Gas permeability coefficients (i.e., O_2_, CO_2_, and H_2_O) of PBF and MCP films were obtained by using a manometric technique. In Fig. 6B, MCP films show superior gas barrier properties with and low gas permeability coefficients (O_2_ 0.320 ~ 0.0187 barrer, CO_2_ and 0.0456 ~ 0.0264 barrer, and H_2_O 3.09 ~ 1.57 × 10^–14^ g cm cm^–2^ s Pa) due to the dense interface structure, high crystallinity and the nanosheets shielding effects (Fig. S15) [17, 49, 50], which arrests the gas penetration and diffusion through the films. Especially, for the MCP3 films, the barrier improvement factors (BIFp, refers to the gas permeability coefficients of virgin PBF divided by the gas permeability coefficient of MCP nanocomposites) of O_2_, CO_2_, and H_2_O are 3.2, 2.8, and 3.0, which are 220% (O_2_ 0.060 barrer), 180% (CO_2_ 0.073 barrer), and 200% (H_2_O 4.70 × 10^–14^ g cm cm^–2^ s Pa) higher than those of virgin PBF, suggesting the importance of MSED structure in enhancing MCP films gas barrier properties. In addition, the water contact angle (Fig. 6B) of the MCP films monotonically increases with increasing filler amounts (Fig. S16). The inert characteristics of CNT and the dense film surface restrict the fast water migration and endow the films with excellent hydrophobic ability [9]. Furthermore, we compare the overall gas barrier performance of MCP films with FDCA-based polyester materials and partial engineering plastics (Fig. 6C). Results demonstrate that our MCP films show the best values for O_2_, CO_2_, and H_2_O (Table S7) due to the nanosheet/crystal double barrier effects in the matrix, confirming its exceptional gas barrier performance. The MCP films with excellent barrier properties offer a brand-new choice for high-performance packaging materials for foods, medicines, and electronic products.

As shown in Fig. 6D and Table S8, MCP films show a high visible light transmittance (550–750 nm) while a low at UV light transmittance (280–400 nm), revealing that the films have superior visible light transmittance and UV resistance (Fig. 6E). Specifically, MCP films can completely impede the UVB (280–320 nm) radiation (Table S8). More significantly, MCP films even filters 85% of UVA (320–400 nm) rays. This optical selectivity is mainly attributed to the uniform dispersion structure of fillers and the photocatalytic effect of MXene itself [22, 24]. When MXene is irradiated by UV lights, which can convert the light energy into heat or other forms for dissipation. Additionally, MCP films also show high solvent resistance. In Fig. 6F, after 30 days immersion in various common solvents (i.e., H_2_O, EtOH, MeOH, 1M NaOH_(aq)_, DMF, and THF) with differing polarities, our MCP films remain their original shapes and appearances, revealing the exceptional durability for chemical solvents of MCP films. Compared with commercial PET and virgin PBF films, the MCP films have both higher tensile strength and higher toughness. Typically, the tensile strength of the MCP films reaches up to 101 MPa, nearly 1.7 times that of PET film (Fig. 6G). Moreover, compared with PET films (O_2_ 0.060 barrer, CO_2_ 0.100 barrer, and H_2_O 3.9 × 10^–14^ g cm cm^–2^ s Pa, details in Table S7), the MCP films can achieve higher gas barrier properties (O_2_ 0.0187 barrer, CO_2_ 0.026 barrer, and H_2_O 1.54 × 10^–14^ g cm cm^–2^ s Pa) and UV resistance (10% vs. 85% UVA). In short, our MCP nanocomposites have superior comprehensive performance, involving ultrahigh strength, toughness, barrier properties, and superb UV shielding performance (Fig. 6G). These integrated exceptional performance enable the MCP nanocomposites promising to be used as green and sustainable engineering plastics and packaging materials for achieving plastic replacement.

## Conclusions

In conclusion, a dendritic hetero-structured MXene@CNT was utilized to realize bio-based PBF polyester nanocomposites with superior mechanical, gas barrier, and functional properties. By reasonable designing and employing the multiple roles of MXene@CNT, i.e., catalyst, nucleator, and interface enhancer of polyesters, the novel MCP nanocomposites were synthesized by in situ catalytic polymerization strategy. Benefited from the multi-scale interfacial interactions, e.g., covalent bonds, hydrogen bonds, mechanical meshing structures, the resultant MCP films show great enhancement in mechanical performance, e.g., ultrahigh tensile strength, stiffness, and toughness reach ≈101 MPa, ≈3.1 GPa, and ≈132 MJ cm^−3^, i.e., about 1.87-, 2.07-, and 1.27-fold higher than that of virgin PBF, respectively, and much higher than most of engineering plastics. More importantly, the dense structure, high crystallization degree, and nanosheets shielding effect were synergistically enhanced the gas barrier properties of MCP; the representative O_2_ barrier property (0.0187 barrer) is 3 times much higher than those of virgin PBF and PET. Additionally, MCP films also have multifunctionality with exceptional physical recycling, ultraviolet resistance, and solvent-resistant performance. Obviously, due to numerous characteristics, e.g., mechanical robustness, superb barrier, excellent reprocessability, superior UV resistance and high solvent resistance, such MCP films show promising applications in engineering plastics and packaging materials for achieving plastic replacement. Importantly, our catalytic-interface strengthening integration strategy provides a brand-new idea for designing and fabricating high-performance polyester materials in future.

## Supplementary Information

Below is the link to the electronic supplementary material.

Supplementary file 1 (DOCX 31302 KB)
