# Supplementary material for: Robust and Reprocessable Biorenewable Polyester Nanocomposites In Situ Catalyzed and Reinforced by Dendritic MXene@CNT Heterostructure
Source: Nanomicro Lett. 2025 Feb 24;17:161. doi: 10.1007/s40820-025-01682-8 (PMC11850687; doi:10.1007/s40820-025-01682-8)
Supplement: Supplementary file 1 — (DOCX 31302 KB) [file 40820_2025_1682_MOESM1_ESM.docx]

Supporting Information for

Robust and Reprocessable Biorenewable Polyester Nanocomposites In-Situ Catalyzed and Reinforced by Dendritic MXene@CNT Heterostructure

Hao Wang^1,2^, Jiheng Ding^1,^*, Hongran Zhao^1^, Qinchao Chu^1,2^, Jin Zhu^1^ and Jinggang Wang^1,^*

^1^Key Laboratory of Bio-based Polymeric Materials Technology and Application of Zhejiang Province, Ningbo Institute of Materials Technology and Engineering, Chinese Academy of Sciences, Ningbo 315201, P. R. China

^2^School of Materials Science and Chemical Engineering, Ningbo University, Ningbo, Zhejiang 315211, P. R. China

*Corresponding authors. E-mail: [dingjh@nimte.ac.cn](mailto:dingjh@nimte.ac.cn) (Jiheng Ding); [wangjg@nimte.ac.cn](mailto:wangjg@nimte.ac.cn) (Jinggang Wang)

**S1 Experimental Section**

**S1.1 Characterizations**

The molecular weight and molecular weight distribution were measured at 40 ^o^C by GPC (Agilent PL-GPC220) equipped with two columns (PLgel 5 mm Mixed-D 300×7.5 mm). Scanning electron microscope (SEM, FEI quanta 250, SE images) and transmission electron microscope (TEM, JEOL JEM2100) were used to analyses the microstructures of samples. Fourier transform infrared spectroscopy (FTIR, Nicolet 6700, Thermo Fisher Scientific, USA), X-ray diffractometer (XRD, D8 Discover/GADDS, Bruker, Germany) with Cu Kα radiation, X-ray photoelectron spectroscopy (XPS, AXIS Ultra DLD, Kratos Analytical, UK), an ultraviolet-visible (UV-Vis, erkinElmer LAMBDA 1050+ UV/Vis/NIR spectrophotometer, UK) with a λ of 200 ~ 800 nm, a Bruker AC-P 400 MHz NMR instrument (Shanghai, China), TGA instrument (Mettler-Toledo TGA/DSC thermogravimetric analysis) were applied to study the chemical structures. Gas transmission rates (PERME VAC-V2, Labthink Instruments Co., Ltd., Jinan, P.R. China) was tested at 23°C and RH 50%, the films with a thickness of about 100 μm were cut into circulars with a diameter of 97 mm and effective permeability area of 38.5 cm^2^. Water vapor transmission rate (PERME W3/060, Labthink Instruments Co., Ltd., Jinan, China) was tested at 38°C and RH 90% with a testing range of 0.1 ~ 10,000 g/m2 24 h, the samples were cut into circulars with a diameter of 33 mm. Small Angle X-ray Scattering (SAXS) was evaluated using a Xeuss 3.0 instrument manufactured by Xenocs, equipped with a Cu target and a Pilatus 300K detector. The distance between the detector and the sample was 600 mm. The differential scanning calorimetry (DSC) analysis was performed on a differential scanning calorimeter (Mettle-Toledo DSC I) to evaluate the thermal properties such as glass transition temperature (T_g_) and melt entropy (ΔH_m_). The samples were heated from -20 °C to 250 °C at a rate of 10 °C/min and held at 250 °C for 3 min, then cooled to -20 °C at a rate of 10 °C/min and reheated to 250 °C at 10 °C/min. According to the ASTM D638, the films were cut into dumbbell-shaped specimens with dimensions of 20 mm (length) × 2 mm (width) × 1 mm (thickness). The mechanical properties were obtained with a universal material testing machine (ZwickRoell tensile tester with the test loads of 1kN, Germany). To ensure the reliability, at least 3 repeated tests were performed.

**S1.2 Theoretical Simulation**

The reaction mechanism and the role of MXene in the polycondensation of PBF were investigated based on density functional theory (DFT) calculations. The DFT-D2 modified van der Waals interactions was also considered during the theoretical simulation. The electron self-consistent iteration is 10^-5^ eV, and all atomic residual forces are below 0.02 eV Å ^-1^. The initial state involved the adsorption of two butylene furandicarboxylate (BFDT) molecules, with a distance of 3.570 Å between the H atom in the furan ring and F in MXene. A vacuum layer along the z-direction was added to avoid periodic interactions. The free energy (GA) was calculated by.

$GA=EA+ZPE-TS (S1)$

where $EA$, $ZPE$, $T$ and $S$ are the total energy, the zero point energy, the temperature (298.15K), and the entropy, respectively.

**S1.3 Supplemental Experimental Procedure**

Isothermal crystallization of the MCP polyester nanocomposite was investigated at different crystallization temperatures and the exothermal plots were tested with a function of crystallization time. The relative crystallinity degree can be calculated if an assumption is done that the change of crystallinity is linearly proportional to the change of heat released in the crystallization process [S1]:

$$X\left( t \right)=\frac{\int_{0}^{t} (dH_{C}/dt)dt}{\int_{0}^{\infty} (dH_{C}/dt)dt} (S2)$$

where $dH_{C}$ is the tested enthalpy of crystallization during an infinitesimal time interval dt. The limits t and ∞ on the integrals are applied to denote the elapsed time during the course of crystallization and at the end of the crystallization process, respectively. Isothermal crystallization kinetics was studied employing the an Avrami approach. The Avrami equation, which posits a constant nucleation rate and constant linear growth, depicts the relative crystallinity degree X(t) in relation to the crystallization time t as follows [S2]:

$$X\left( t \right)=1-exp(-kt^{n}) (S3)$$

where n is the Avrami exponent, which is a function of the nucleation process, and k is the growth function, which depends on nucleation and crystal growth. The values of n and k can be calculated from the fitting to experimental results employing the double logarithmic form of Eq. S4 [S3].

$$log\left\{ -ln\left[ 1-X_{(t)} \right] \right\}=nlogt+logk (S4)$$

Eq. S4 was applied to fit the experimental results of isothermal melt crystallization of MCP. 𝑙𝑜𝑔{−𝑙𝑛[1 − 𝑋(𝑡)] was plotted against log t. After fitting the linear part, the slope and the intersect of the Avrami plots were employed to estimate the data of n and k, respectively. Only the first linear parts were employed for the fittings because the Avrami is commonly valid until the primary crystallization ends.

**S2 Supplementary Figures and Tables**

**
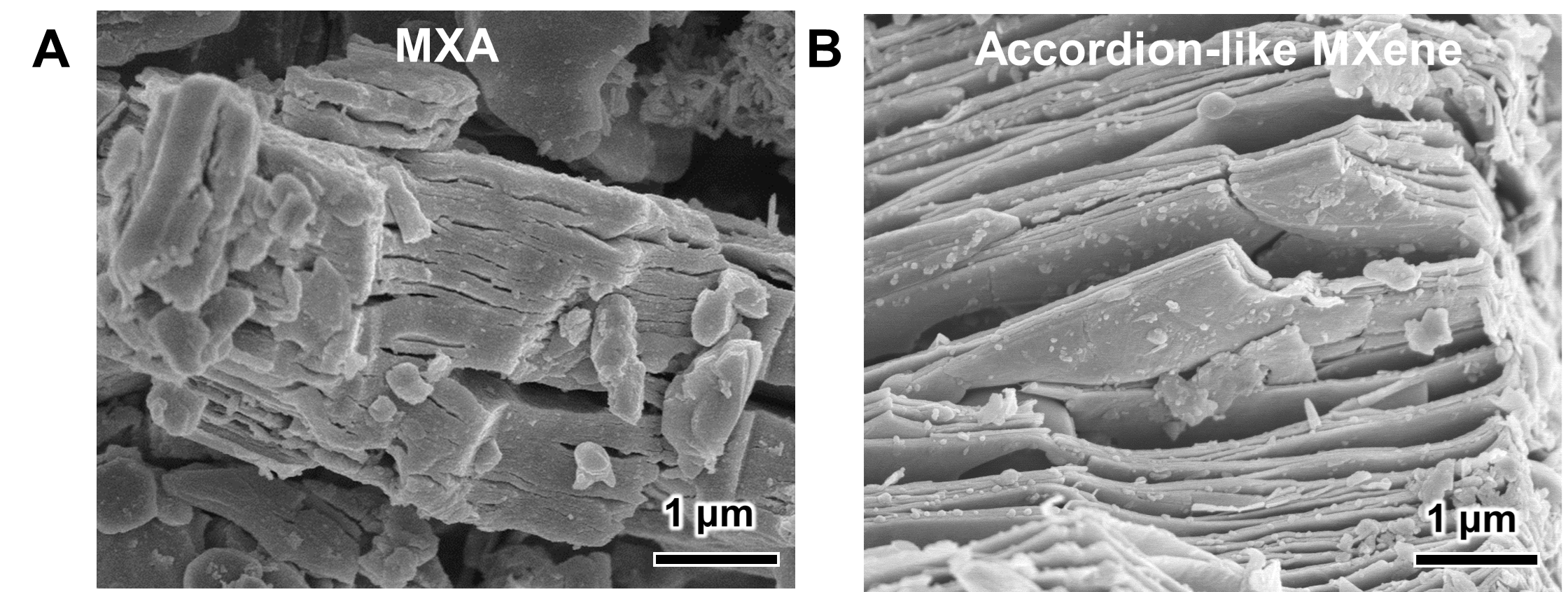
**

**Fig. S1** SEM images of Ti_3_AlC_2_ (**A**) and accordion-like Ti_3_C_2_T_x_ MXene (**B**)


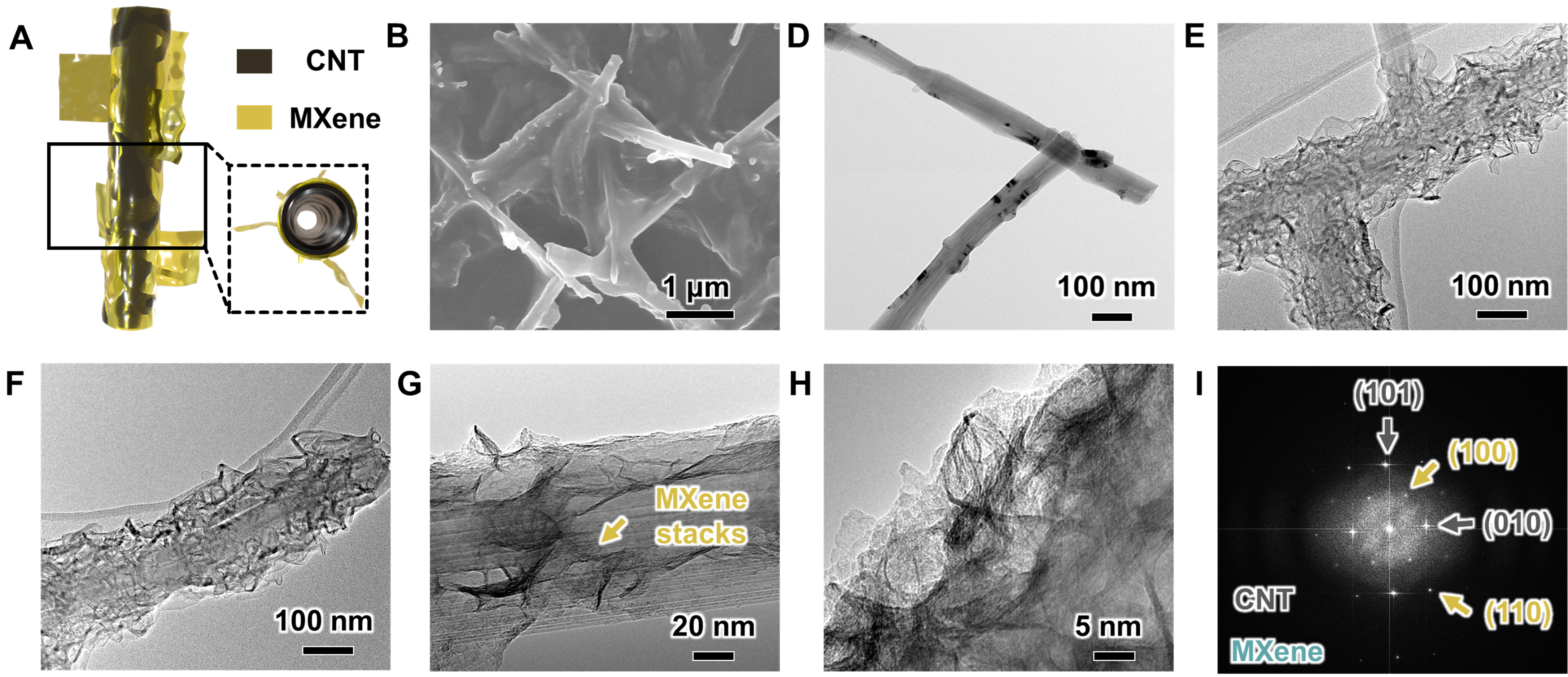


**Fig. S2** Schematic diagram of the multi-scale MXene@CNT sample (**A**). SEM image of MXene@CNT dispersed on Si substrate (**B**). TEM images of CNT (**C**) and MXene@CNT (**E-G**). High-resolution TEM images (**H**) and corresponding FFT patterns **(I**) of MXene@CNT


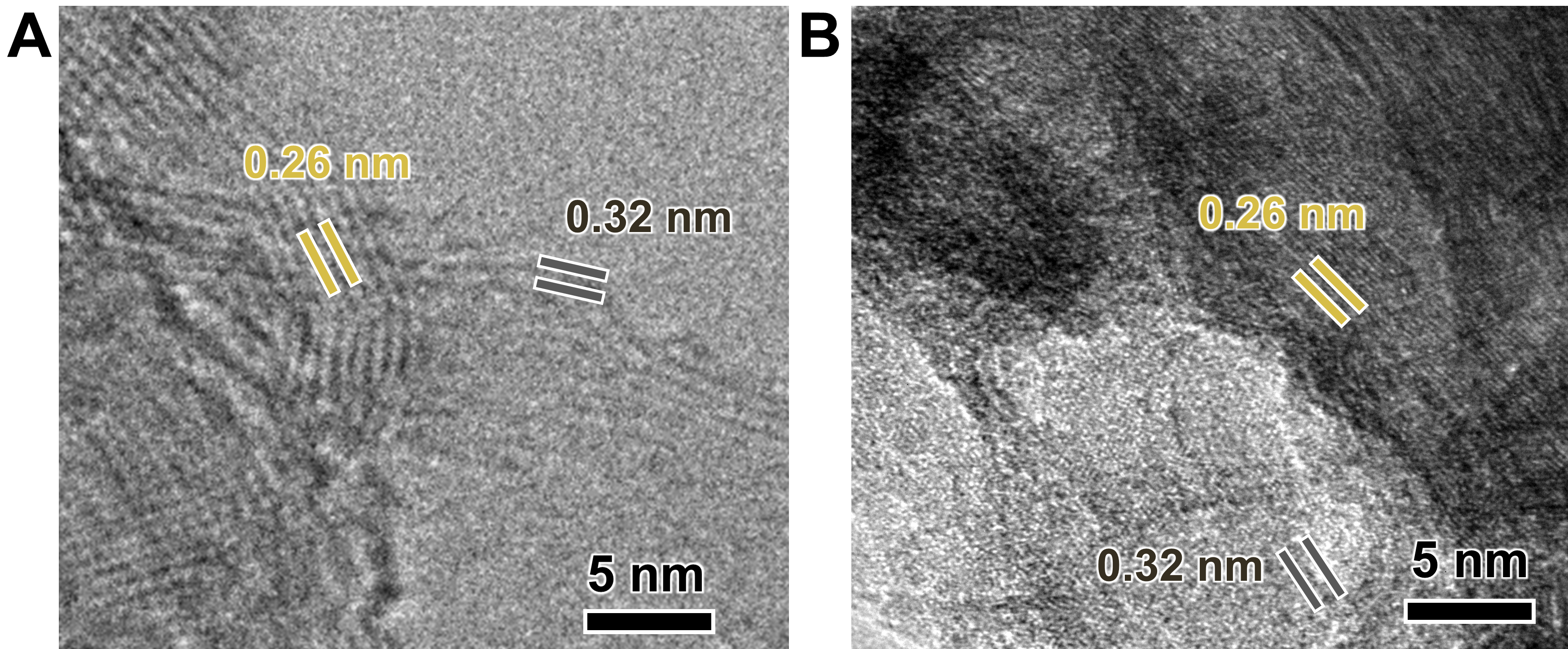


**Fig. S3** HRTEM images of the junction between MXene nanosheets and CNT


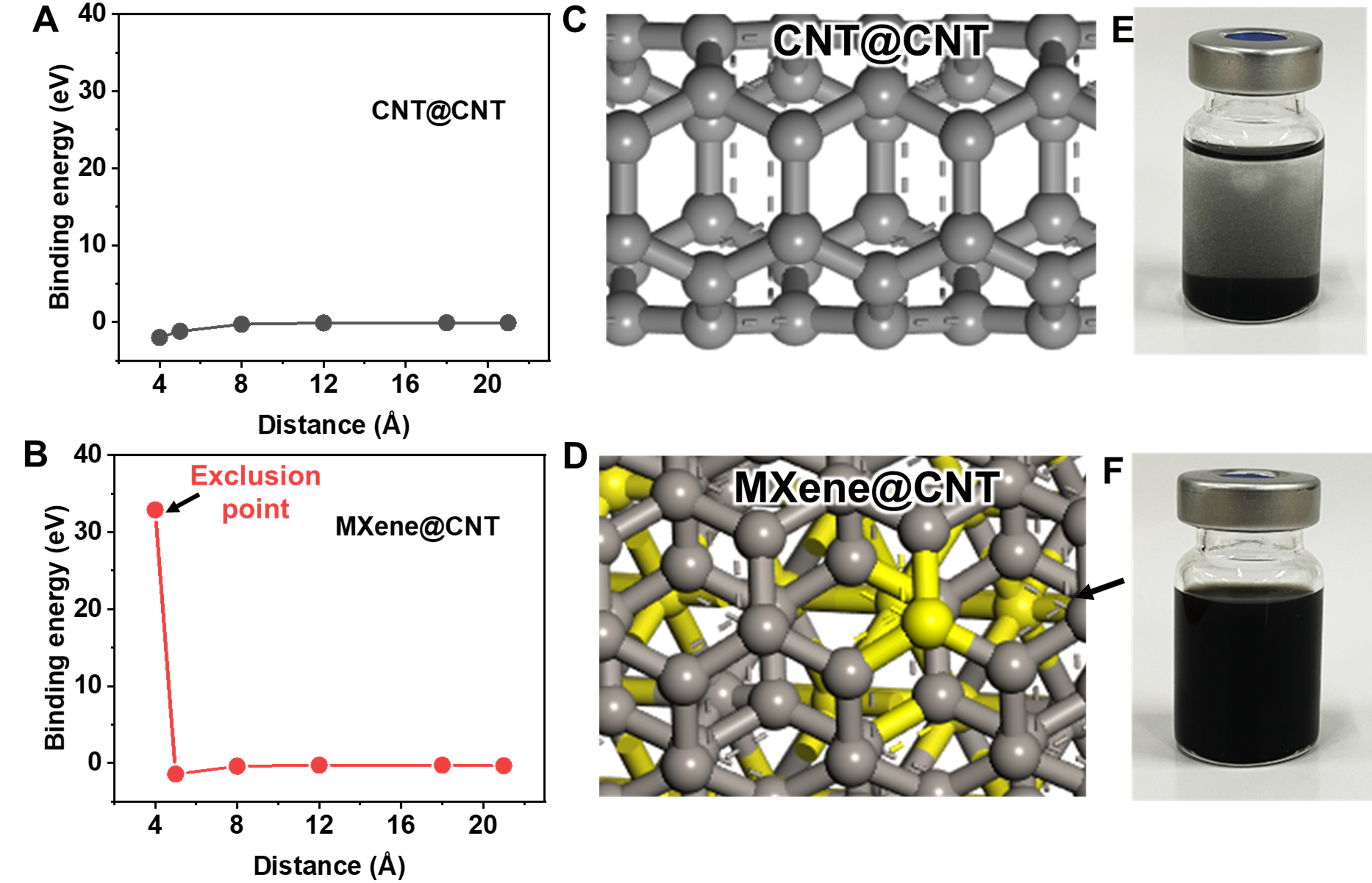


**Fig. S4** Binding energy as the layer distance of CNT@CNT (**A**) and MXene@CNT (**B**) hybrids based on DFT calculation and corresponding optimal geometric models (**C, D**). The initial atomic configuration consists of an individual CNT with a diameter of 10 Å and monolayered MXene nanosheets. The interaction between CNT and MXene is only described by van der Waals forces [Lennard-Jones (LJ) potential]. Digital photos of CNT (**E**) and MXene@CNT (**F**) BDO dispersions after storge for 1 week


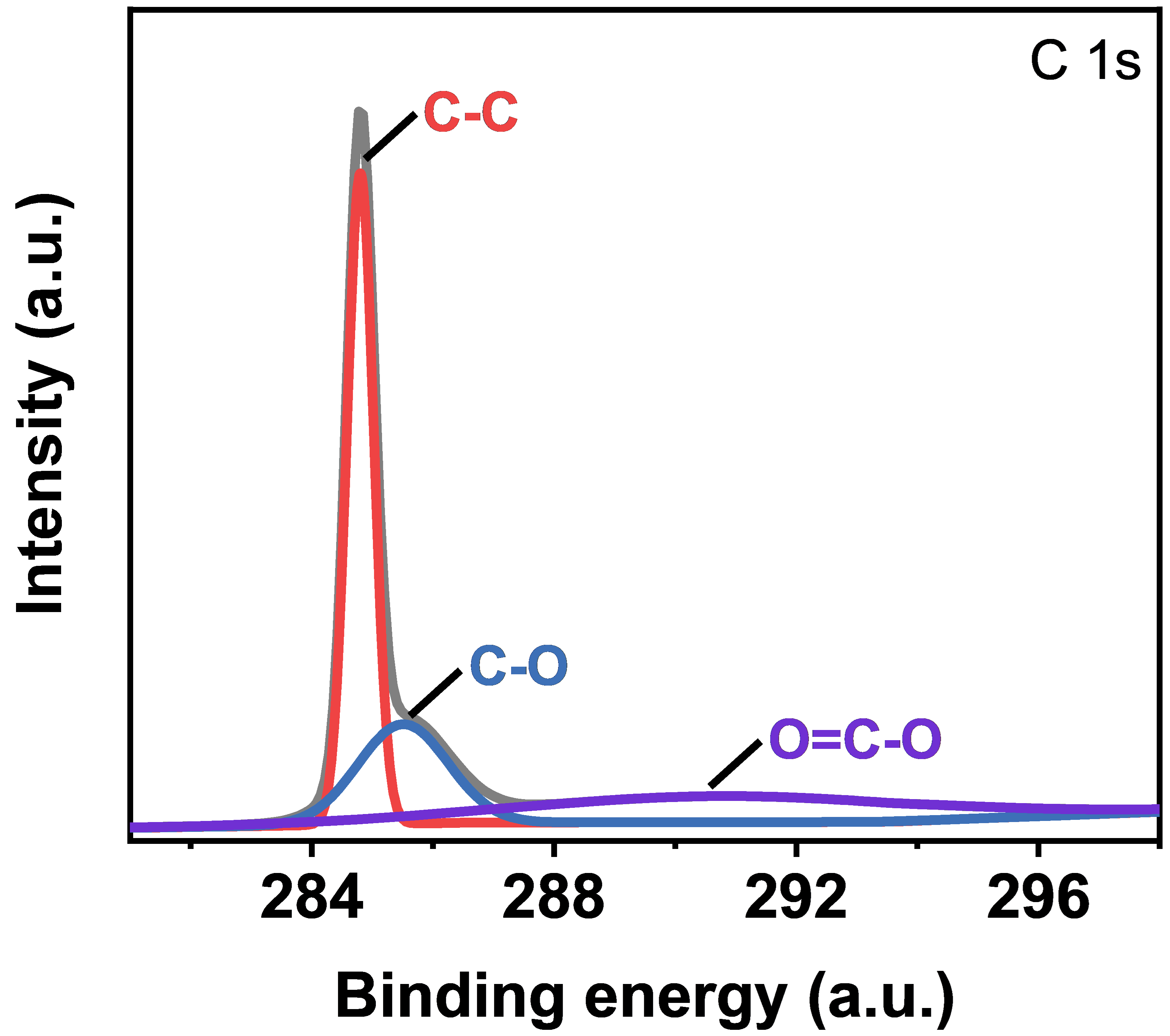


**Fig. S5** XPS C_1s_ fine spectra of CNT, in which the residual C-O and COOH functional groups are easily detected


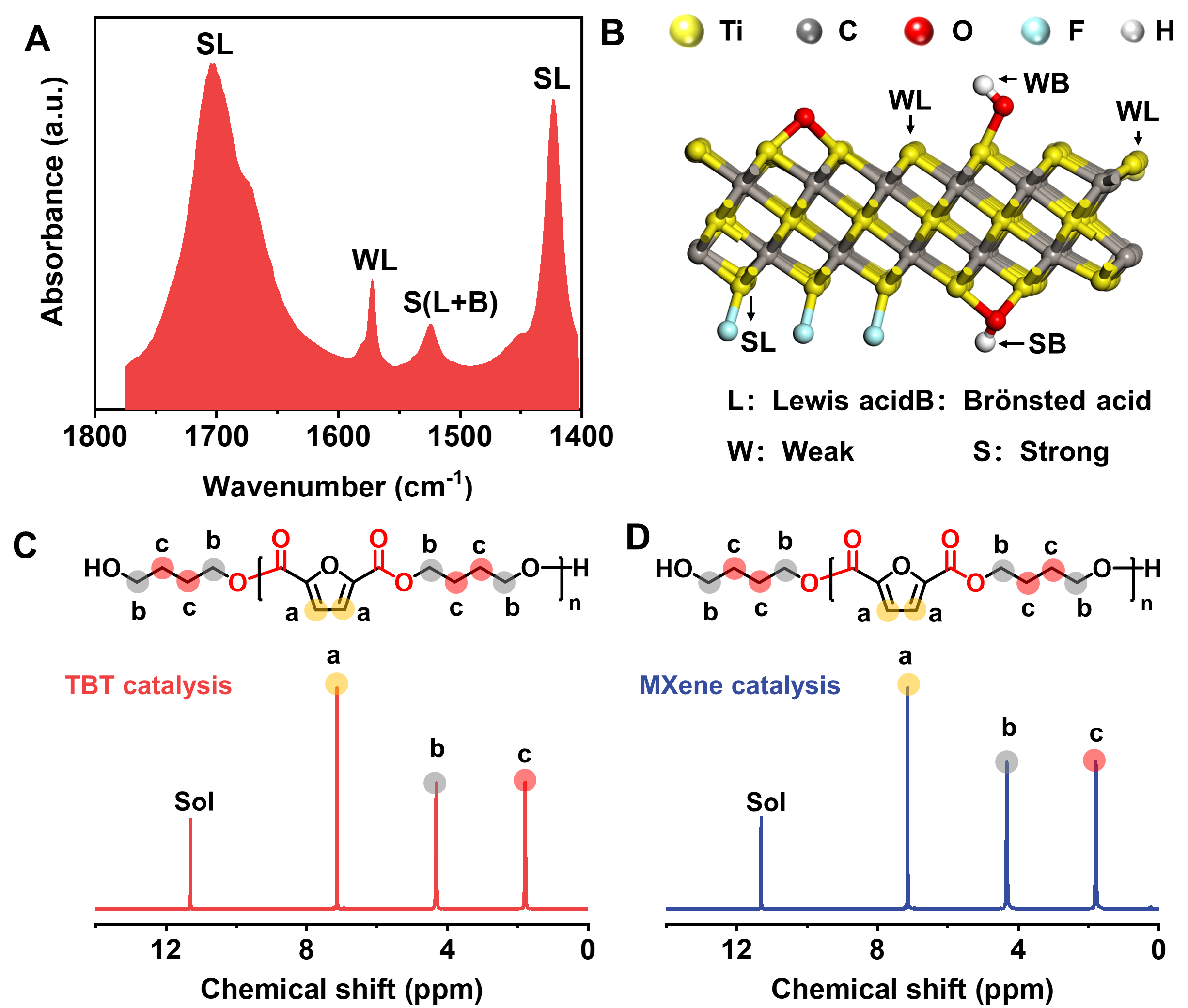


**Fig. S6** Pyridine DRIFTS for MXene (**A**). Illustration of acid site types on MXene revealing stronger sites close to F groups and weaker sites from O/OH groups (**B**) [S4]. H^1^ NMR spectra of PBF synthesized utilizing TBT (**C**) and MXene (D) as catalysts


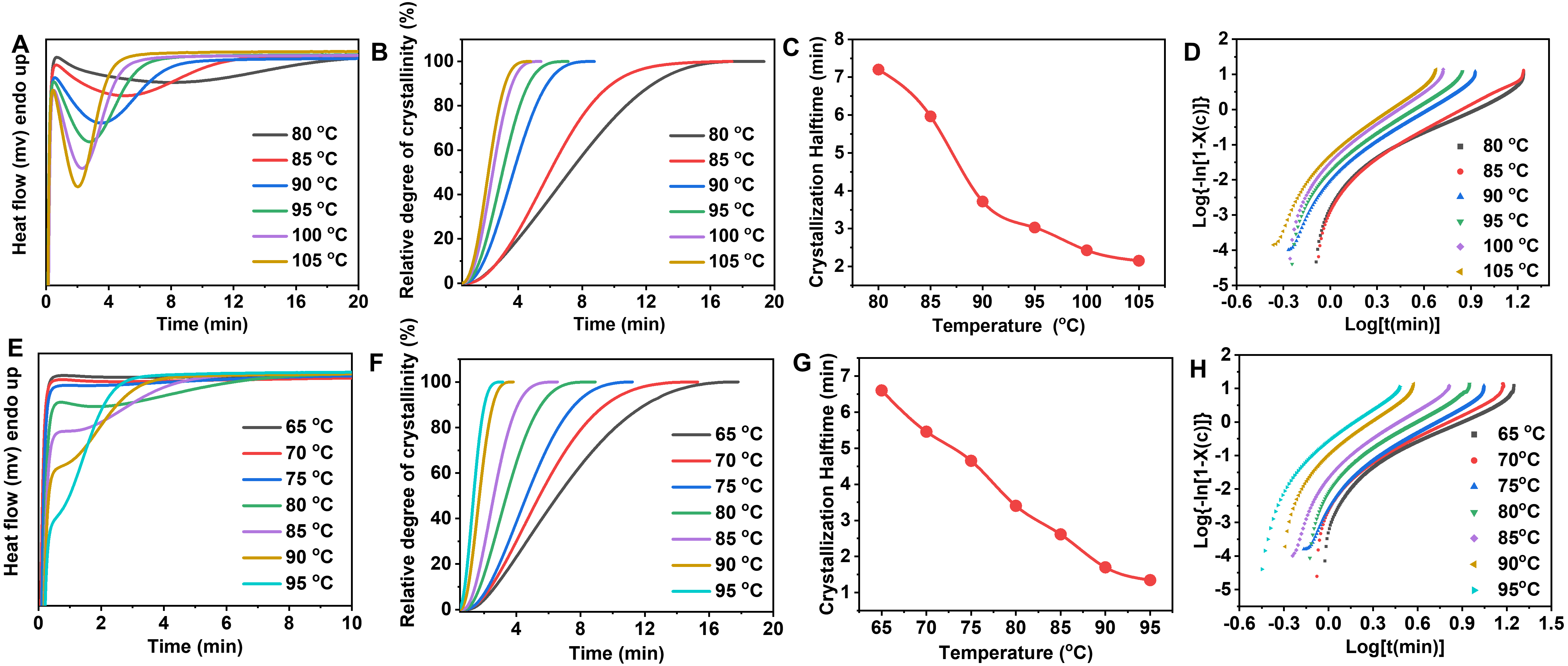


**Fig. S7** Isothermal crystallization peaks recorded at the indicated temperatures for PBF (**A**) and MCP (**E**). Evolution of the relative degree of crystallinity with time for PBF (**B**) and MCP (**F**). Halftime of crystallization versus the isothermal crystallization temperature for PBF (**C**) and MCP (**G**). Avrami plots for the isothermal crystallization for PBF (**D**) and MCP (H)


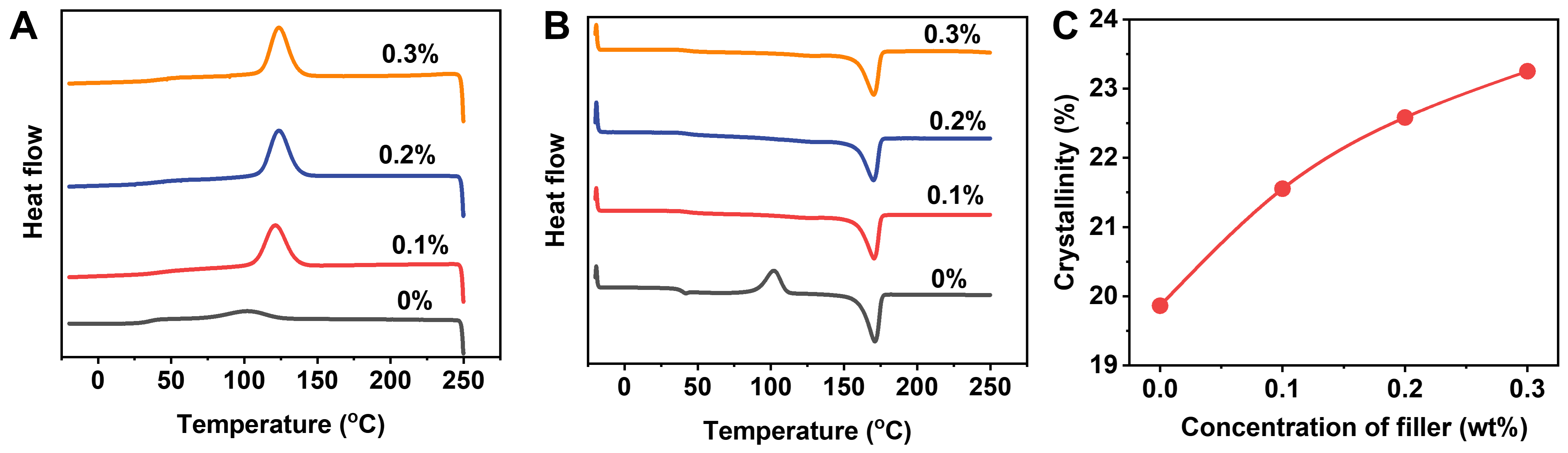


**Fig. S8** DSC curves of MCP with different filler content (**A, B**). The changes of crystallinity with the filler content (**C**)


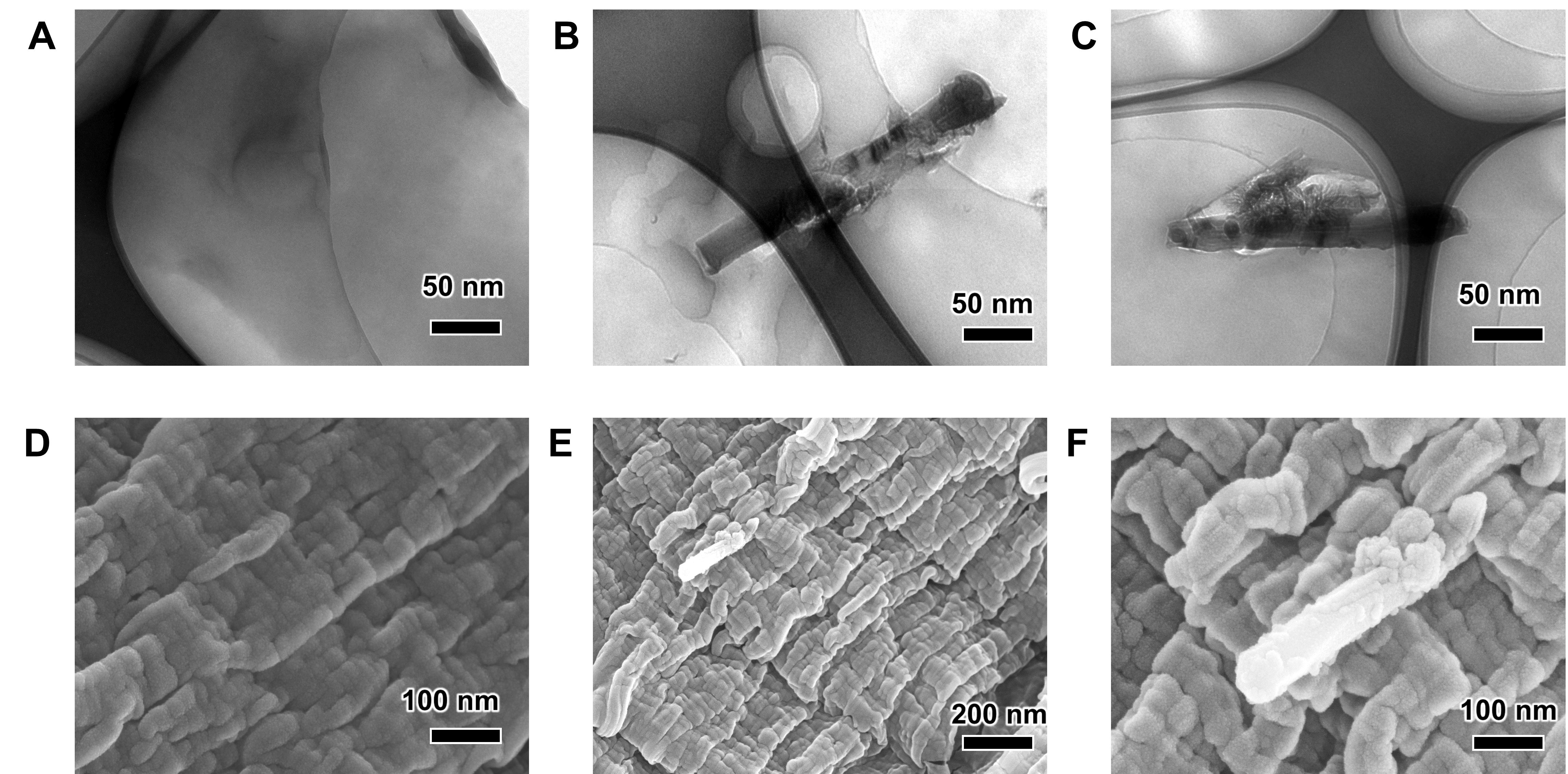


**Fig. S9** Cross-section TEM images of PBF (**A**) and MCP (**B, C**). Cross-section SEM images of PBF (**D**) and MCP nanocomposites (**E, F**)


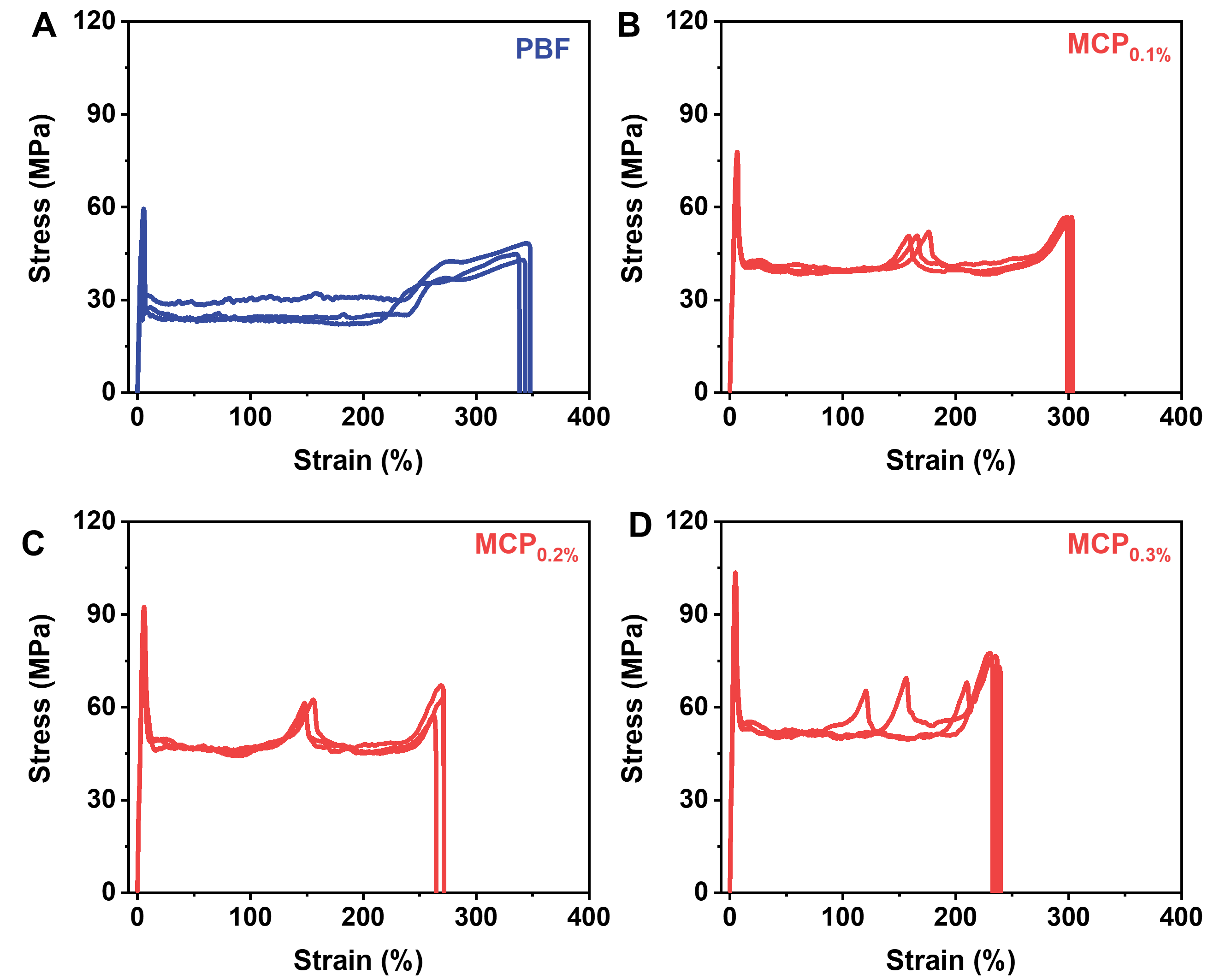


**Fig. S10** Stress-strain plots of PBF (**A**), MCP_0.1%_ (**B**), MCP_0.2%_ (**C**), and MCP_0.3%_ (**D**)


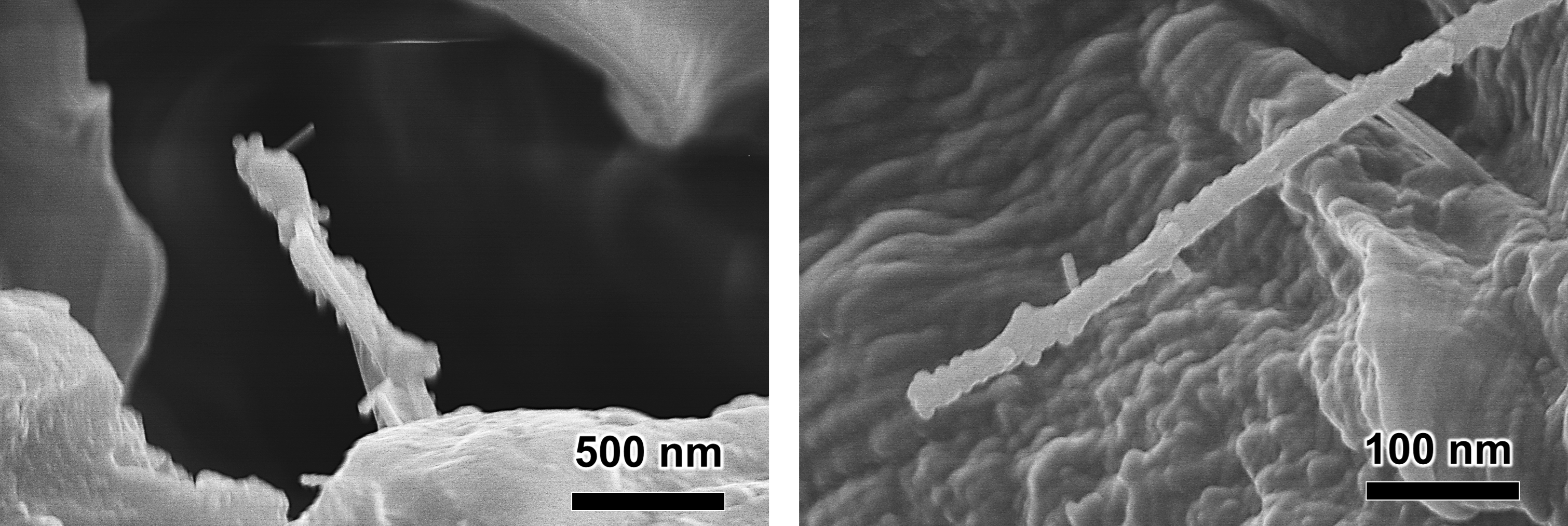


**Fig. S11** Cross-sectional SEM images of MCP after fracture


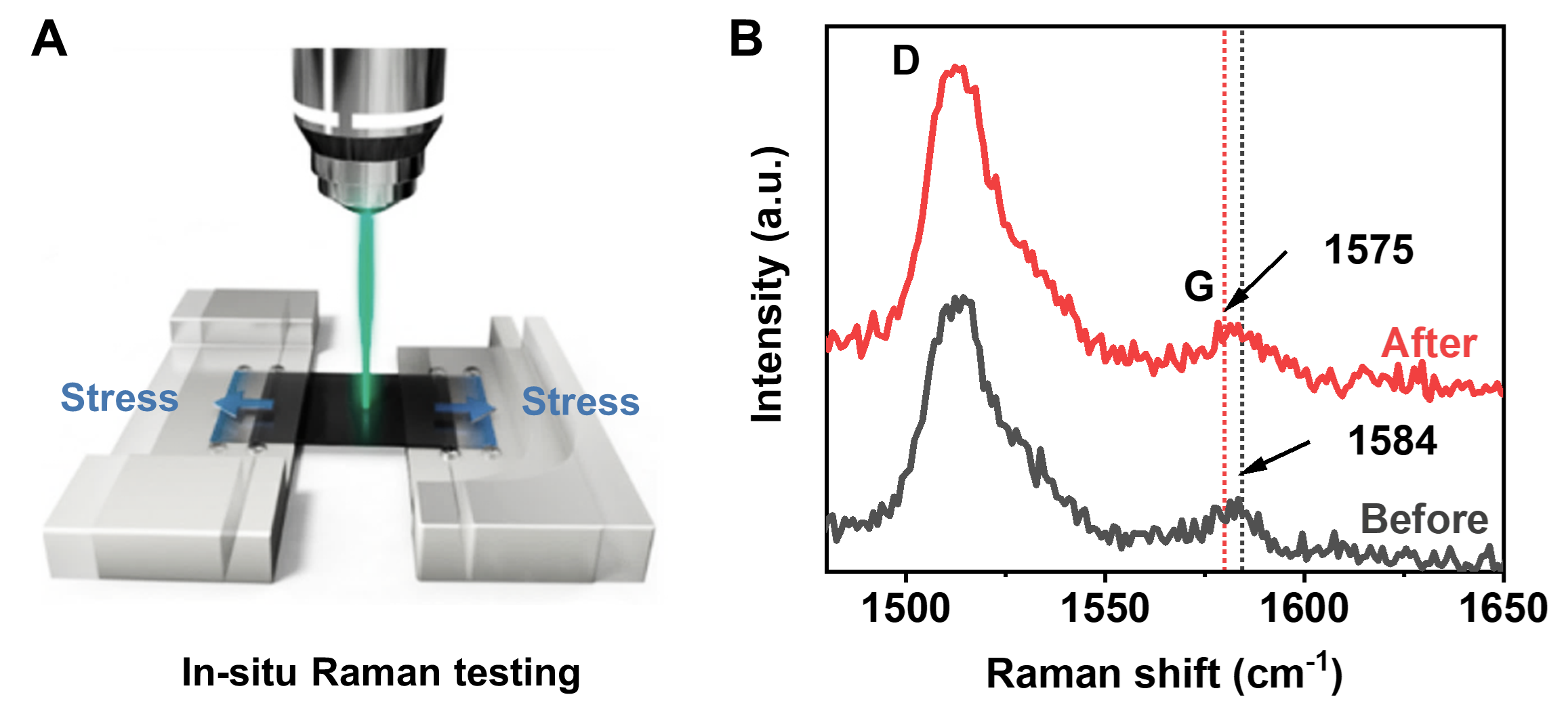


**Fig. S12** Schematic diagram of in-situ Raman testing (**A**) [S5]. Changes of G band of CNT in MCP system during the stretching (**B**)


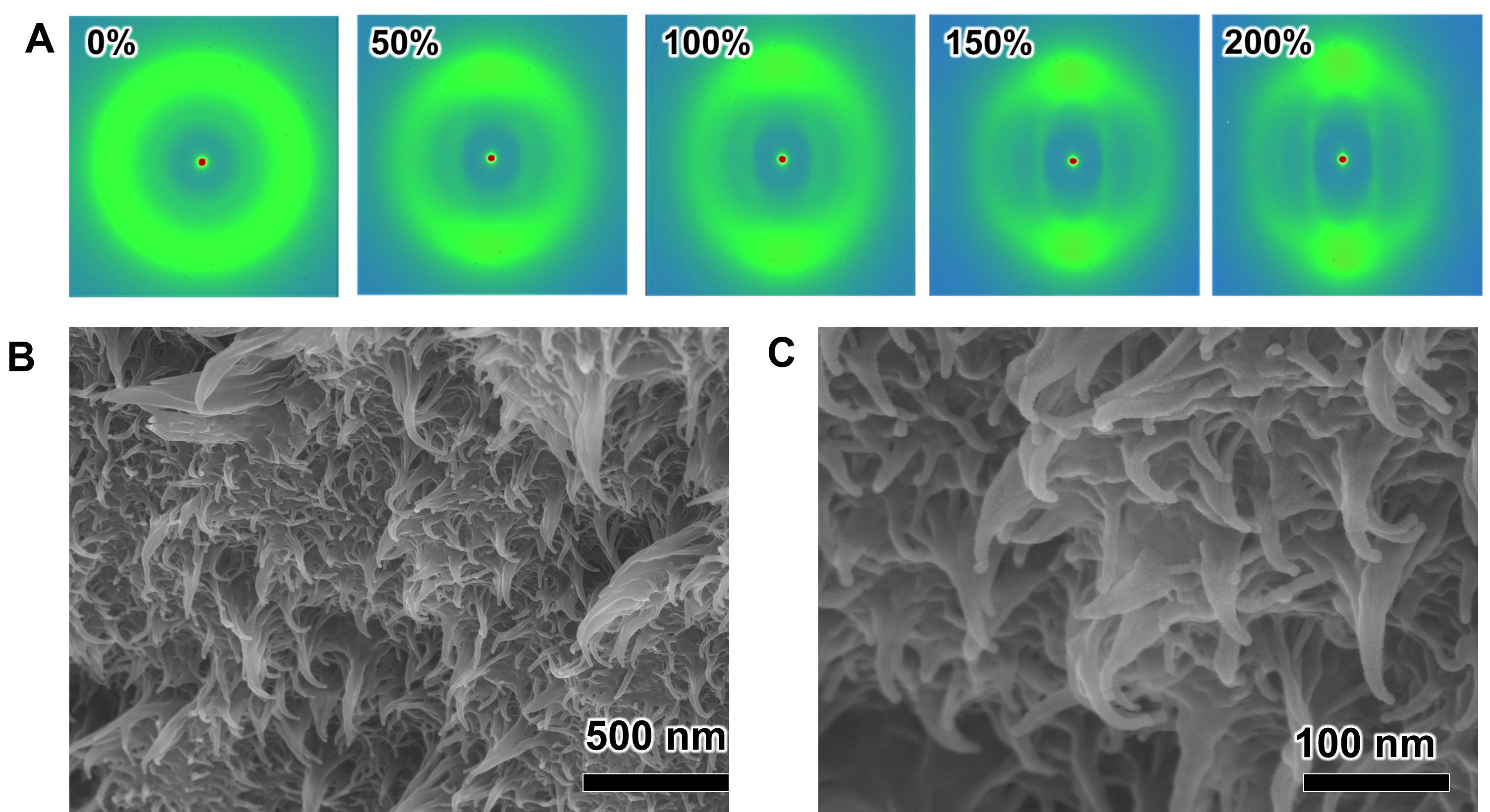


**Fig. S13** 2D WAXS patterns during the stretching (**A**), revealing the stress-induced orientation of PBF chains. Cross-sectional SEM images of MCP after fracture (**B, C**)


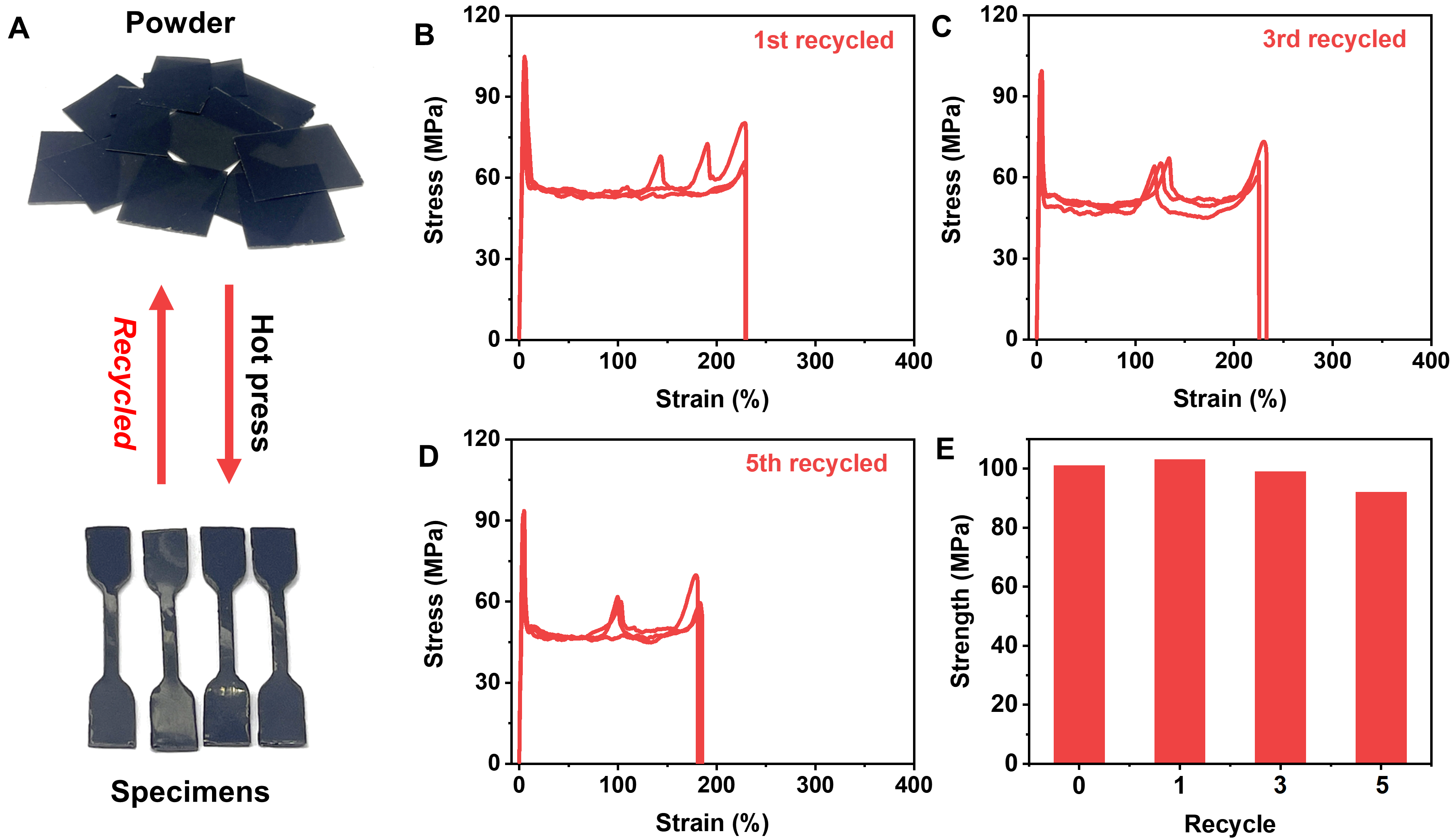


**Fig. S14** Physical reprocessing of MCP polyester nanocomposites (**A**). Stress-strain curves of MCP after 1^st^ (**B**), 3^rd^ (**C**), and 5^th^ (**D**) recycled. Comparison of tensile strength of MCP in different cycles (**E**)


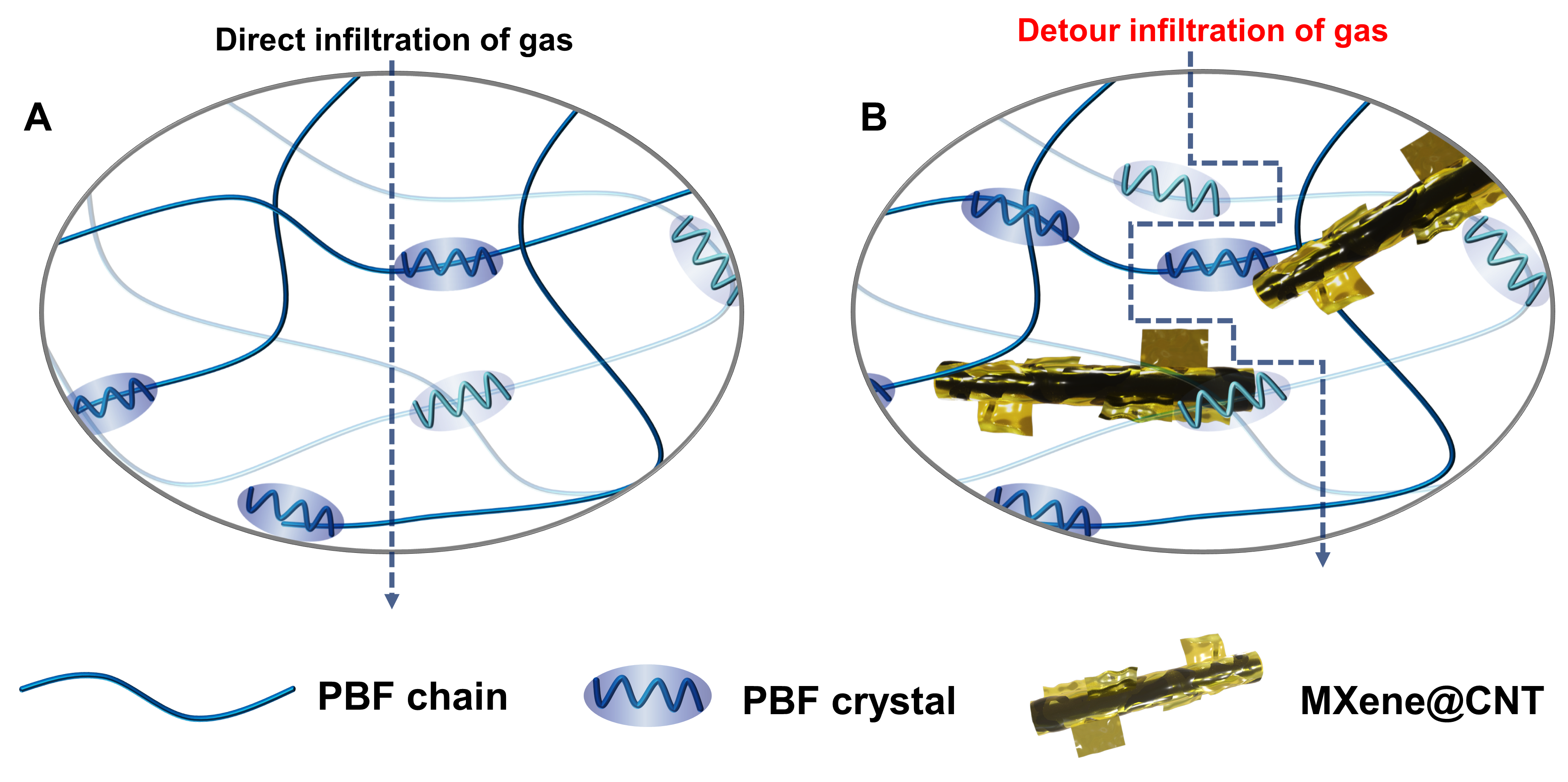


**Fig. S15** Gas barrier mechanisms of PBF (**A**) and MCP (**B**). Due to the poor physical barrier effect of pure polymers allows gas to quickly penetrate the PBF membrane, thus resulting in a high permeability coefficient. For the MCP, the gas barrier mechanism can be attributed to the follow [S6, S7]: 1) The higher polymer crystals and MXene lamellae maximizes the physical barrier effect; 2) The fillers increased the free volume, making gas transport difficult; 3) MCP has a strong interface, making it difficult for gas to penetrate quickly


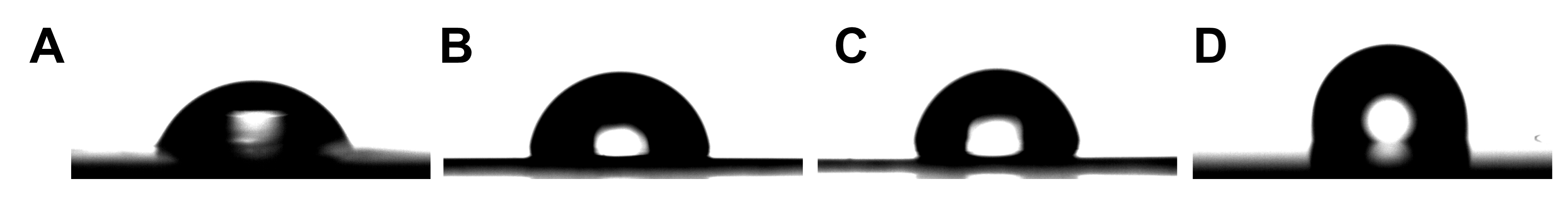


**Fig. S16** WCA values of PBF (**A**), MCP_0.1%_ (**B**), MCP_0.2%_ (**C**), and MCP_0.1%_ (**D**)

**Table S1** Interaction energy of hybrids with the distance of layers

| **Hetero-structured Type** | ***Distance* (Å)** | **E (eV)** |
| --- | --- | --- |
| CNT@CNT | 21 | -0.07 |
|  | 18 | -0.07 |
|  | 12 | -0.10 |
|  | 8 | -0.26 |
|  | 5 | -1.17 |
|  | 4 | -1.99 |
| MXene@CNT | 21 | -0.34 |
|  | 18 | -0.27 |
|  | 12 | -0.27 |
|  | 8 | -0.41 |
|  | 5 | -1.42 |
|  | 4 | 32.89 |

**Table S2** Molecular weight and intrinsic viscosity of different polyester materials

| **Sample** | **Filler** | **M_n_ (g/mol)** | **M_w_ (g/mol)** | **PD** | **[η_sp_](dl/g)** |
| --- | --- | --- | --- | --- | --- |
| PBF | 0.0 wt% | 4.2×10^4^ | 7.1×10^4^ | 1.67 | 0.94 |
| MCP | 0.1 wt% | 4.0×10^4^ | 7.0×10^4^ | 1.77 | 0.97 |
| MCP | 0.2 wt% | 4.1×10^4^ | 7.1×10^4^ | 1.74 | 0.97 |
| MCP | 0.3 wt% | 4.3×10^4^ | 7.6×10^4^ | 1.76 | 1.05 |

**Table S3** Mechanical properties parameters of different polyester materials

| **Sample** | **Filler** | **σ_t_^a^ (MPa)** | ***E*^a^ (GPa)** | **ε^a^ (%)** | **τ^a^ (MJ/m^3^)** |
| --- | --- | --- | --- | --- | --- |
| PBF | 0.0 wt% | 54 ± 6 | 1.5 ± 0.1 | 343 ± 8 | 104 ± 5 |
| MCP | 0.1 wt% | 79 ± 1 | 1.8 ± 0.1 | 301 ± 2 | 127 ± 1 |
| MCP | 0.2 wt% | 91 ± 1 | 2.4 ± 0.1 | 270 ± 3 | 132 ± 2 |
| MCP | 0.3 wt% | 101 ± 2 | 3.1 ± 0.1 | 237 ± 2 | 130 ± 2 |

**Notes:** ^a^σ_t_, E, *ε*, and *τ* refer to tensile strength, Young’s modulus, elongation at break, and tensile toughness, respectively.

**Table S4** Mechanical performances of reported FDCA-based polyesters

| ***No*.** | **Tpye** | **Sample** | **σ_t_ (MPa)** | **ε (%)** | ***E* (GPa)** | **Refs.** |
| --- | --- | --- | --- | --- | --- | --- |
| 1 | FDCA-  based  *homo*-  polyester | PET | 60 | 110 | 2.2 | [*S8*] |
| 2 |  | PEF | 52 | 2.3 | 2.58 | [*S9*] |
| 3 |  | PBF | 54 | 343 | 1.5 | This work |
| 4 |  | PHF | 35.5 | 210 | 0.5 | [*S10*] |
| 5 |  | POF | 20.3 | 15 | 0.34 | [*S10*] |
| 6 |  | PCF | 62 | 18 | 2.1 | [*S11*] |
| 7 | FDCA-  based  *co*-  polyester | PECF | 58 | 120 | 2.2 | [*S11*] |
| 8 |  | PBCF | 59 | 5 | 1.4 | [*S12*] |
| 9 |  | PEPeF | 22 | 286 | 1.7 | [*S13*] |
| 10 |  | PPeCBT | 38 | 68 | 1.96 | [*S14*] |
| 11 |  | PPSF | 48 | 196 | 1.24 | [*S15*] |
| 12 |  | PETF | 66 | 85 | 1.12 | [*S16*] |
| 13 | FDCA-  based  polyester  composites | PHF/CNT | 18.9 | 80 | 0.65 | [*S17*] |
| 14 |  | PHF/CCF | 9.3 | 188 | 0.4 | [*S18*] |
| 15 |  | PEF/MNS | 67 | 5.5 | 3.1 | [*S19*] |
| 16 |  | PEF/CNT | 80 | 2.5 | 4.12 | [*S20*] |
| 17 |  | PEF/BNNS | 79 | 2.1 | 4.2 | [*S20*] |
| 18 |  | PBF/BNNS-CNT | 76 | 193 | 2.3 | [*S8*] |
| 19 |  | PEF/BNNS-LDH | 140 | 2.4 | 6.5 | [*S9*] |
| **20** |  | **MCP** | **101** | **237** | **3.1** | **This work** |

**Table S5** Mechanical properties parameters of different cycled polyester materials

| **Sample** | **σ_t_ (MPa)** | ***E* (GPa)** | **ε (%)** | **τ (MJ/m^3^)** |
| --- | --- | --- | --- | --- |
| 1^st^ recycled | 102 ± 1 | 3.1 ± 0.1 | 229 ± 2 | 128 ± 1 |
| 3^rd^ recycled | 99 ± 1 | 3.0 ± 0.1 | 228 ± 4 | 119 ± 1 |
| 5^th^ recycled | 92 ± 1 | 3.0 ± 0.1 | 184 ± 2 | 91 ± 2 |

**Table S6** Gas barrier properties parameters of different polyester films

| **Sample** | **Filler**  **(wt%)** | **O_2_^a^ (barrer)** | **BIFp** | **CO_2_^a^**  **(barrer)** | **BIFp** | **H_2_O^b^** | **BIFp** |
| --- | --- | --- | --- | --- | --- | --- | --- |
| PBF | 0.0 | 0.0600 | 1.0 | 0.0730 | 1.0 | 4.70 | 1.0 |
| MCP | 0.1 | 0.0320 | 1.9 | 0.0456 | 1.6 | 3.09 | 1.5 |
| MCP | 0.2 | 0.0296 | 2.0 | 0.0334 | 2.2 | 2.26 | 2.1 |
| MCP | 0.3 | 0.0187 | 3.2 | 0.0264 | 2.8 | 1.57 | 3.0 |

**Notes:** ^a^O_2_ and CO_2_ permeability coefficient, at 23 °C, 50% test was carried out at 0.1001 MPa, 23 ^o^C, 50% relative humidity, 1 barrer = 10^-10^ cm^3^ cm/cm^2^·s·cm Hg. ^b^H_2_O permeability coefficient, at 38 ^o^C, 90% relative humidity, 10^-14^ g cm/cm^2^·s·Pa.

**Table S7** Gas permeability coefficients for MCP and other commercial plastics

| ***NO.*** | **Type** | **Sample** | **O_2_^a^**  **(Barrer)** | **BIFp** | **CO_2_^a^**  **(Barrer)** | **BIFp** | **H_2_O**  **(g/(m^2^ d)** | **H_2_O**  **(g·cm/(cm^2^ s Pa)×10^-14^** | **BIFp** | **Refs.** |
| --- | --- | --- | --- | --- | --- | --- | --- | --- | --- | --- |
| 1 | FDCA-  based  *homo*-  polyester | PEF | 0.020 | 1 | 0.04 | 1 | / | 1.64 | 1 | (*S8*) |
| 2 |  | PBF | 0.021 | 0.95 | 0.0385 | 1.04 | / | 4.488 | 0.36 | This work |
| 3 |  | PNF | 0.035 | 0.57 | 0.04 | 1 | / | 2.58 | 0.635 | [*S22*] |
| 4 | FDCA-  based  *co*-  polyester | PBCF_40_ | 0.10 | 0.2 | 0.70 | 0.057 | / | 13 | 0.0126 | [*S23*] |
| 5 |  | PBCF_50_ | 0.046 | 0.43 | 0.30 | 0.13 | / | 6.6 | 0.248 | [*S23*] |
| 6 |  | PBCF_60_ | 0.037 | 0.54 | 0.25 | 0.16 | / | 4.3 | 0.38 | [*S23*] |
| 7 |  | PBF_40_-PEG | 3.7 | 0.005 | 19.3 | 0.002 | / | 721 | 0.0022 | [*S24*] |
| 8 |  | PBF_50_-PEG | 2.1 | 0.009 | 12.1 | 0.003 | / | 563 | 0.0029 | [*S24*] |
| 9 |  | PBF_60_-PEG | 1.2 | 0.017 | 6.9 | 0.006 | / | 303 | 0.0054 | [*S24*] |
| 10 |  | PBF_70_-PEG | 0.56 | 0.037 | 3.2 | 0.0062 | / | 144 | 0.0114 | [*S24*] |
| 11 |  | PBF_80_-PEG | 0.078 | 0.256 | 0.18 | 0.11 | / | 41.3 | 0.040 | [*S24*] |
| 12 |  | PBF_90_-PEG | 0.024 | 0.83 | 0.030 | 1.33 | / | 15.9 | 0.103 | [*S24*] |
| 13 |  | PPSF_40_ | 0.04 | 0.5 | 0.60 | 0.067 | / | 9.37 | 0.175 | [*S15*] |
| 14 |  | PPSF_50_ | 0.027 | 0.74 | 0.57 | 0.07 | / | 6.37 | 0.257 | [*S15*] |
| 15 |  | PNSF_40_ | 0.042 | 0.48 | 0.053 | 0.75 | / | 4.83 | 0.339 | [*S22*] |
| 16 |  | PNSF_60_ | 0.024 | 0.83 | 0.030 | 1.33 | / | 4.78 | 0.343 | [*S22*] |
| 17 |  | PBFLA_40_ | 0.033 | 0.61 | 0.14 | 0.285 | / | 3.7 | 0.443 | [*S25*] |
| 18 | Petro-based  plastics | PE | 1.580 | 0.013 | 6.29 | 0.006 | 7.9 | / | 0.232 | [*S26]* |
| 19 |  | PP | 2.750 | 0.007 | 3.82 | 0.001 | 2.2 | / | 0.837 | [*S27*] |
| 20 |  | PLA | 0.250 | 0.08 | 1.00 | 0.04 | / | 11 | 0.149 | [*S15*] |
| 21 |  | PBAT | 0.760 | 0.026 | 5.90 | 0.0068 | / | 35.2 | 0.046 | [*S25*] |
| 22 |  | PET | 0.06 | 0.26 | 0.1 | 0.4 | / | 3.9 | 0.42 | [*S8*] |
| **23** | Composite polyester | **MCP** | **0.0187** | **1.11** | **0.026** | **1.54** | **/** | **1.57** | **1.044** | **This work** |

**Notes:** ^a^O_2_ and CO_2_ permeability coefficient, at 23 °C, 50% test was carried out at 0.1001 MPa, 23 ^o^C, 50% relative humidity, 1 barrer = 10^-10^ cm^3^ cm/cm^2^·s·cm Hg.

**Table S8** UV shielding and visible light transmittance parameters of polyester films

| **Sample** | **UVC shielding**  **(280-320 nm)** | **UVB shielding**  **(280-320 nm)** | **UVA shielding**  **(320-400 nm)** | **Visible light transmittance**  **(550-750 nm)** |
| --- | --- | --- | --- | --- |
| PBF | 100% | 90% | 20% | 88% |
| MCP1 | 100% | 94% | 53% | 67% |
| MCP2 | 100% | 100% | 65% | 65% |
| MCP3 | 100% | 100% | 85% | 60% |

**Supplementary References**

1. H. Wang, X. Shi, Y. Xie, S. Gao, Y. Dai et al., A furan-containing biomimetic multiphase structure for strong and supertough sustainable adhesives. Cell Rep. Phy. Sci. **4**, 101374 (2023). <https://doi.org/10.1016/j.xcrp.2023.101374>
2. L. Sangroniz, B. Wang, Y. Su, G. Liu, D. Cavallo et al., Fractionated crystallization in semicrystallinepolymers. Prog. Polym. Sci. **115**, 101376 (2021). <https://doi.org/10.1016/j.progpolymsci.2021.101376>
3. Y. Zheng, P. Pan, Crystallization of biodegradable and biobased polyesters: polymorphism, cocrystallization, and structure-property relationship. Prog. Polym. Sci*.* **109**, 101291 (2020). <https://doi.org/10.1016/j.progpolymsci.2020.101291>
4. X. Fei, Y. Wang, P. Guo, J. Wang, G. Wang et al., Efficient catalytic activity of Ti_3_C_2_T_x_ MXene for polyester synthesis. Ind. Eng. Chem. Res. **63**, 6868-6879 (2024). <https://doi.org/10.1021/acs.iecr.3c04485>
5. J. Y. Oh, Y. S. Kim, Y. Jung, S. J. Yang, C. R. Park, Preparation and exceptional mechanical properties of bone-mimicking size-tuned graphene oxide@carbon nanotube hybrid paper. ACS Nano **10**, 2184-2192 (2016). <https://doi.org/10.1021/acsnano.5b06719>
6. X. Fei, J. Wang, J. Zhu, X. Wang, X. Liu, Biobased poly(ethylene 2,5-furancoate): no longer an alternative, but an irreplaceable polyester in the polymer industry. ACS Sustainable Chem. Eng. **8**, 8471-8485 (2020). <https://doi.org/10.1021/acssuschemeng.0c01862>
7. Y. Shi, C. Chen, Y. Li, W. Zhao, Achieving dual functional corrosion resistance for epoxy coatings under alternating hydrostatic pressure via constructing p-phenylenediamine/Ti_3_C_2_T_x_ hybrids. Carbon **201**, 1048-1060 (2023). <https://doi.org/10.1016/j.carbon.2022.09.089>
8. J. Ding, H. Zhao, H. Wang, Q. Chu, J. Zhu et al., Flexible and recyclable bio-based polyester composite films with outstanding mechanical and gas barrier properties using leaf-shaped CNT@BNNS covalent heterojunction. Small 2406958 (2024). <https://doi.org/10.1002/smll.202406958>
9. J. Ding, H. Zhao, S. Shi, J. Su, Q. Chu et al., High-strength, high-barrier bio-based polyester nanocomposite films by binary multiscale boron nitride nanosheets. Adv. Funct. Mater. 2308631 (2023). <https://doi.org/10.1002/adfm.202308631>
10. M. Jiang, Q. Liu, Q. Zhang, C. Ye, G. A Zhou, Series of furan-aromatic polyesters synthesized via direct esterification method based on renewable resources. J. Polym. Sci. A: Polym. Chem. ***50 (5)***, 1026-1036 (2021). <https://doi.org/10.1002/pola.25859>
11. J. Wang, X. Liu, Y. Zhang, F. Liu, J. Zhu, Modification of poly(ethylene 2,5-furandicarboxylate) with 1,4-cyclohexanedimethylene: influence of composition on mechanical and barrier properties. Polymer ***103***, 1-8 (2016). <https://doi.org/10.1016/j.polymer.2016.09.030>
12. X. Fei, Y. Zhu, J. Wang, Z. Jia, X. Liu, Synthesis of bio-based polyesters with crystallization properties comparable to poly(butylene terephthalate). Polym. Adv. Technol*.* ***33***, 2265-2275 (2022). <https://doi.org/10.1002/pat.5677>
13. H. Xie, L. Wu, B.-G. Li, P. Dubois, Modification of poly(ethylene 2,5-furandicarboxylate) with biobased 1,5-pentanediol: significantly toughened copolyesters retaining high tensile strength and O_2_ barrier property. Biomacromolecules ***20***, 353-364 (2019). <https://doi.org/10.1021/acs.biomac.8b01495>
14. Y. Chen, L. Wu, B.-G. Li, Poly(1,5-pentylene-co-2,2,4,4-tetramethyl cyclobutylene terephthalate) copolyesters with high T_g_ and improved ductility and thermal stability. Polymer ***232***, 124152 (2021). <https://doi.org/10.1016/j.polymer.2021.124152>
15. H. Hu, R. Zhang, J. Wang, W. B. Ying, J. Zhu, Fully bio-based poly(propylene succinate-co-propylene furandicarboxylate) copolyesters with proper mechanical, degradation and barrier properties for green packaging applications. Eur. Polym. J*.* ***102***, 101-110 (2018). <https://doi.org/10.1016/j.eurpolymj.2018.03.009>
16. G. Wang, X. Hao, M. Jiang, R. Wang, Y. Liang et al., Partially bio-based copolyesters poly(ethylene 2,5-thiophenedicarboxylate-co-ethylene terephthal- ate): synthesis and properties. Polym. Degrad. Stabil. ***181***, 109369 (2020). <https://doi.org/10.1016/j.polymdegradstab.2020.109369>
17. M. Chen, Z. Jiang, Z. Qiu, In situ synthesis, crystallization behavior, and mechanical property of biobased poly(hexamethylene 2,5-furandicarboxylate)/ multiwalled carbon nanotube nanocomposites. Ind. Eng. Chem. Res. ***61***, 9745-9754 (2022). <https://doi.org/10.1021/acs.iecr.2c01449>
18. S. Pan, Z. Jiang, Z. Qiu, Crystallization and mechanical property of fully biobased poly (hexamethylene 2,5-furandicarboxylate)/cellulose nanocrystals composites. Polymer ***267****,* 125689 (2023). <https://doi.org/10.1016/j.polymer.2023.125689>
19. J. Ding, H. Wang, H. Zhao, S. Shi, J. Su et al., Large-size ultrathin mica nanosheets: reinforcements of biobased PEF polyester. Giant ***18***, 100264 (2024). <https://doi.org/10.1016/j.giant.2024.100264>
20. H. Wang, J. Ding, H. Zhao, Q. Chu, M. R. Miah et al., Preparing strong, tough, and high-barrier biobased polyester composites by regulating interfaces of carbon nanotubes. Mater. Today Nano ***25***, 100463 (2024). <https://doi.org/10.1016/j.mtnano.2024.100463>
21. M. R. Miah, J. Ding, H. Zhao, Q. Chu, H. Wang et al., Boron nitride-based polyester nanocomposite films with enhanced barrier and mechanical performances. ACS Appl. Polym. Mater. ***6***, 2913-2923 (2024). <https://doi.org/10.1021/acsapm.3c03148>
22. H. Hu, R. Zhang, Y. Jiang, L. Shi, J. Wang et al., Toward biobased, biodegradable, and smart barrier packaging material: modification of poly(neopentyl glycol 2,5- furandicarboxylate) with succinic acid. ACS Sustainable Chem. Eng. **7**, 4255-4265 (2019). <https://doi.org/10.1021/acssuschemeng.8b05990>
23. H. Hu, R. Zhang, J. Wang, W. B. Ying, J. Zhu, Synthesis and structure-property relationship of biobased biodegradable poly(butylene carbonate-co-furan- dicarboxylate). ACS Sustainable Chem. Eng. **6**, 7488-7498 (2018). <https://doi.org/10.1021/acssuschemeng.8b00174>
24. H. Hua, R. Zhang, A. Sousac, Y. Long, W. B. Ying et al., Bio-based poly(butylene 2,5-furandicarboxylate)-b-poly(ethylene glycol) copolymers with adjustable degradation rate and mechanical properties: synthesis and characterization. Eur. Polym. J. **106**, 42-45 (2018). <https://doi.org/10.1016/j.eurpolymj.2018.07.007>
25. H. Hu, R. Zhang, L. Shi, W. B. Ying, J. Wang et al., Modification of poly(butylene 2,5-furandicarboxylate) with lactic acid for biodegradable copolyesters with good mechanical and barrier properties. Ind. Eng. Chem. Res. **57**, 11020-11030 (2018). <https://doi.org/10.1021/acs.iecr.8b02169>
26. L. Marangoni Júnior, R.M.V. Alves, C.Q. Moreira, M. Cristianini, M. Padula et al., High-pressure processing effects on the barrier properties of flexible packaging materials. J. Food Process. Preserv. **44**, e14865 (2020). <https://doi.org/10.1111/jfpp.14865>
27. J. Yi, Y. Li, Y. Zhao, Z. Xu, Y. Wu, M. Jiang, G. Zhou, Development of A Series of Biobased Poly(ethylene 2,5-furandicarboxylate-*co*-(5,5′-((phenethyl- azanediyl) -bis(methylene))bis(furan-5,2-diyl))dimethylene 2,5-furandi- carboxylate) Copoly-mers via A Sustainable and Mild Route: Promising “Breathing” Food Packaging Materials. Green Chem*.* **24**, 5181-5190 (2022). <https://doi.org/10.1039/D2GC01214F>
